# Transcriptome-based analysis reveals key molecular mechanisms and functional characterization of *MaCAX3* gene involved in manganese stress responses in mulberry plants

**DOI:** 10.1186/s12870-025-06767-5

**Published:** 2025-07-29

**Authors:** Jianbin Li, Michael Ackah, Frank Kwarteng Amoako, Aaron Tettey Asare, Manman Shen, Zhenjiang Wang, Qiang Lin, Changyu Qiu, Meina Zhu, Mengdi Zhao, Weiguo Zhao

**Affiliations:** 1 Jiangsu Key Laboratory of Sericulture Biology and Biotechnology, School of Biotechnology, Jiangsu University of Science and Technology, Zhenjiang, 212100 People’s Republic of China; 2 Key Laboratory of Silkworm and Mulberry Genetic Improvement, Ministry of Agriculture and Rural Affairs, The Sericultural Research Institute, Chinese Academy of Agricultural Sciences, Zhenjiang, 212100 People’s Republic of China; 3 Institute of Plant Nutrition and Soil Science, Kiel University, Hermann- Rodewald-Straße 2, 24118 Kiel, Germany; 4 Department of Molecular Biology and Biotechnology, School of Biological Sciences, College of Agriculture and Natural Sciences, University of Cape Coast, PMB Ghana, Cape Coast, Ghana; 5 Sericulture & Agri-Food Research Institute, Guangdong Academy of Agricultural Sciences, Guangzhou, 510610 China; 6 Sericulture Technology Promotion Station, Guangxi Zhuang Autonomous Region, Nanning, 530007 China; 7 Department of Materials Science and Engineering, Suzhou University of Science and Technology, Suzhou, 215011 P. R. China

**Keywords:** *Morus alba*, Manganese, Transcriptome, Gene silencing, Cell wall polysaccharides, *MaCAX3* gene, Reactive oxygen species

## Abstract

**Background:**

Manganese (Mn) deficiency and toxicity are major constraints on crop production in soil. Plants have evolved cascade strategies and specific mechanisms to tolerate these stresses. Understanding the molecular mechanisms of tolerance to Mn stress is crucial for improving the efficiency of conferring Mn tolerance and phytoremediation, which is intriguing for evolutionary research on plant adaptation to abiotic stresses. In this study, the responses of mulberry to varied concentration levels of Mn (MnSO_4_), ranging from deficiency (0 mM and 0.03 mM), sufficiency (0.15 mM), and toxicity regimes (1.5 mM and 3 mM) were compared by elucidating the physiological, transcriptome profiling, and functional characterization of the *MaCAX3* gene in mulberry leaves.

**Results:**

The results show that Mn-induced deficiency and toxicity not only trigger an increase in oxidation and antioxidant parameters, including hydrogen peroxide (H_2_O_2_), lipid peroxidase (LPO), polyphenol oxidase (PPO), and reactive oxygen species (ROS) but also concomitantly improved the activities of total antioxidant capacity (TAC) and hydroxyl radical (•OH) scavenging levels in mulberry. Results of the cell wall structural components show that cellulose, hemicellulose, and lignin contents were significantly higher, except for pectin, in the control (CK) compared to the deficiency and toxicity. Functional validation of the *MaCAX3* gene via gene silencing revealed that the heterologous expression of the *MaCAX3* gene increased the transport of Mn in yeast, thus inhibiting the toxic effect of Mn relative to the silenced *Macax3*-VIGS. Additionally, transcriptome analysis identified a total of 811 differentially expressed genes (DEGs), with 189 and 622 being up- and downregulated, respectively. These DEGs were significantly involved in Mn transport, detoxification, oxidation, antioxidant defense, and cell wall and protein processing, which conferred tolerance to Mn in mulberry plants.

**Conclusion:**

The study sheds substantial light on key molecular mechanisms and the functional characterization and validation of crucial Mn tolerance genes in mulberry leaves.

**Supplementary Information:**

The online version contains supplementary material available at 10.1186/s12870-025-06767-5.

## Introduction

Manganese (Mn) is a trace element with immeasurable and great significance for plant growth and development. Apart from its functional roles and participation in cascades of metabolic processes including photosynthesis, respiration, secondary metabolism, and protein biosynthesis, it also forms an indispensable component of the Mn-cluster structure in plant cells [1–3]. Mn not only participates in metabolic processes but also acts as a major cofactor of a series of enzymes, including superoxide dismutase (SOD), catalase (CAT), enzymes involved in the tricarboxylic acid cycle (TCA cycle) such as phosphoenolpyruvate carboxylase (PEPC) and flavonoid and lignin biosynthesis in plants [1, 4, 5]. Research has proven time without number that the lack and excessive supply of Mn disrupts both the catabolism and anabolism processes of plants. That means any Mn amount below or above the optimal levels has a great tendency to cause growth impairment in plants. For instance, relative to deficiency and toxicity, the normal amount (control) of Mn supplied to mulberry plants increased fresh and dried biomass by 36% and 54%, and 28% and 56%, respectively [1], indicating the detrimental consequences of Mn deficiency and toxicity in the plant. This phenomenon highlights the need to urgently find adaptive crops, and any necessary efforts toward improving and championing plants to cope and adapt to Mn stresses is a worthy cause.

To counteract Mn deficiency and toxicity and its ripple effects, plants have evolved sophisticated and versatile strategies to detoxify Mn, including modification of Mn translocation and distribution within plant cells, sequestration and subcellular compartmentalization of Mn, activation of antioxidant biosynthesis systems, and doubling the chelation of Mn through organic acid exudation [2, 3]. Physiologically, several results evident that reactive oxygen species (ROS) scavenging systems involving antioxidant enzymes, including peroxidase (POD) and ascorbate peroxidase (APX), are modulated and regulated by the plant’s response to lack and excess Mn in plants such as mulberry (*Morus alba* L.) [1], thereby alleviating Mn-induced oxidative stress in plants [2, 6]. Additionally, secondary metabolic processes and metabolites, including phenolics, flavonoids, and phenylalanine are highly regulated in plants upon Mn stress, highlighting the crucial roles of secondary metabolites in plants adaptation to Mn stresses. For instance, in the flavonoid biosynthesis pathway, metabolite classes such as coumarins and derivatives, isoflavonoids, phenol ethers, and phenols were found to be significantly downregulated when mulberry leaves were exposed to diverse levels of Mn concentration [1].

Plants cope with Mn stress via uptake, translocation, and distribution to assist in ameliorating the ripple effects of Mn stress. To some extent, certain key genes involved in uptake, translocation, and distribution have been characterized and are known to participate in detoxifying diverse Mn stresses. Recently, genes such as the *Fe–SOD* (iron superoxide dismutase) have been reported to contribute to Mn toxicity tolerance in perennial ryegrass [7], *OsMTP8.1* and *OsMTP8.2* identified to be involved in the sequestration of Mn into vacuoles to adapt to Mn toxicity [8] in rice (*Oryza sativa*) and, *AtECA1* and *AtECA3* were reported to move excess Mn into the endoplasmic reticulum and Golgi apparatus in Arabidopsis [9]. Similarly, the natural resistance-associated macrophage protein (Nramp) members (*OsNramp3*) in rice were identified as plasma membrane Mn transporters for the distribution, mobilization, and translocation of Mn from young leaves to older tissues for the alleviation of Mn toxicity [10]. Furthermore, recent studies have revealed numerous genes involved in the sequestration of Mn into the vacuole for possible detoxification and reuse during deficiency. These detoxification genes belong to the metal tolerance proteins (MTPs) of the cation diffusion facilitator (CDF) family and include the *ShMTP1* reported in the Caribbean stylo plants (*Stylosanthes hamata*) [11], *AtMTP8* identified in Arabidopsis [12], *CsMTP8* found in cucumber (*Cucumis sativus*) [13] and *CasMTP8* characterized in the tea plant (*Camellia sinensis*) [14]. Notwithstanding, Modareszadeh et al. reported that the over-expression of the *CAX3* (encoding a cation/proton exchanger) gene promotes Cd tolerance by reducing ROS (reactive oxygen species) production through the triggering of Ca level in Arabidopsis. *CAX* genes are involved in the sequestration of metal ions (Mn, Li, Cd, and Ca) from the cytosol into vacuole using proton gradients for final detoxification [15]. Even though the crucial roles of some of the above-mentioned genes have functionally been characterized, however, the transcriptome profiling of Mn-responsive genes in mulberry and many other plants is not fully annotated or elucidated and requires urgent attention. Hence, studying the responses of plants to varying Mn concentrations is useful to evaluate how mulberry plants adapt to Mn deficiency and toxicity.

Mulberry is a perennial woody plant with the potential to remediate contaminated soils, highlighting its usefulness in plant phytoremediation [16]. Mulberry plants have been revealed to have superior tolerance to Mn, Cd, Li, B, etc [17–19]. It has recently been documented that high Mn adaptability capabilities in mulberry is attained by its fine-tuned co-regulation of genes and metabolites involved in specific pathways, including flavonoid biosynthesis, defense response and metabolism [1, 18]. Although mulberry has considerable capabilities for Mn tolerance, the molecular responses of mulberry to Mn stress remain largely unknown, especially the effects of Mn deficiency and excess on profile alterations in gene expression and functional characterization of the *CAX3* gene in mulberry have not been reported. To the best of our knowledge, this is the first study to unravel the molecular mechanism of mulberry Mn stress tolerance via transcriptomics and gene silencing. However, previous studies have laid the foundations for the current study, which is dissecting the molecular mechanisms and functional responses of mulberry to Mn deficiency and toxicity. Accordingly, in this study, the effects of various Mn concentrations on the physiological and molecular mechanisms of mulberry were investigated. In parallel, the transcriptomic and functional characterization of *MaCAX3* gene performed using an RNA-seq approach and gene silencing reveals the upregulation of *CAX3* gene in mulberry leaves, highlighting that *CAX3* plays a crucial role in Mn tolerance in mulberry. It must be noted that this study is based on the premise of our previous study [1] and that all morpho-physiological data and other relevant parameters are published. The results of this study provide a platform to understand and shed light on the adaptive responses of mulberry to Mn deficiency and toxicity in the pathways and genes involved in Mn tolerance.

## Materials and methods

### Mulberry plant material, growth conditions, Mn treatments, and growth parameters

We obtained the mulberry (Yu-711) from the National Mulberry GenBank at Jiangsu University of Science and Technology, Zhenjiang, Jiangsu, China. We conducted the growth experiment in a controlled greenhouse environment, cultivating the seedlings in vermiculite. Methods outlined by Zhang et al. (2023) were utilized in the cultivation of mulberry seedlings. The treatment of mulberry seedlings with MnSO_4_ and sampling of leaves followed the same procedure as outlined in our previous study on Mn [1] after 21 days (d) of treatment. These leaves exhibited clear deficiency symptoms and were subsequently stored in a freezer at -80℃. For each experimental group, a combined sample of nine leaves was collected, with three (three biological replicates) leaves taken from each mulberry plant. This study is based on our previous study [1], where parameters such as growth (fresh and dry weight, root length), mineral analysis (Mn contents in root and leaf), photosynthesis (net photosynthesis rate, stomatal conductance, intercellular CO_2_, and transpiration rate) were determined and have laid the foundation of this current study to avoid repetition and duplication of results. The same plant samples for the previous study were used in this present study.

### Determination of oxidation and antioxidants and cell wall structure components and their enzymatic activities

Biochemical and physiological parameters, including chlorophyll content (Chl *a*, *b*), proline (PRO), soluble protein, soluble sugar, malondialdehyde (MDA), peroxidase (POD), superoxide dismutase (SOD), and catalase (CAT) have been determined [1] and have laid the foundation of this current study. The determination of oxidation and antioxidants indicator, cell wall structure component, and their enzymatic activity were carried out from mulberry leaves in all groups treated with different concentrations of MnSO_4_ on the 21 d of treatment. To evaluate the content of hydrogen peroxide (H_2_O_2_), cellulose, hemicellulose, and lignin contents, reactive oxygen species (ROS) production rate, total antioxidant capacity (TAC), hydroxyl radical (•OH) scavenging rate, and enzyme activity of lipid peroxidase (LPO), polyphenol oxidase (PPO), cellulase, xylanase and pectinase were detected using Kit provided by Suzhou Keming Biotechnology Co., Ltd, Suzhou, China. The preparation of mulberry leaf samples after the Mn treatments followed strictly the instructions provided in the detection Kit and three biological and technical replicates were used in each indicator. Data was processed using a one-way ANOVA and means were separated using Tukey at *p* ≤ 0.05 and all statistical analysis was conducted in R software (v4.2). Figures were plotted in GraphPad Prism (v10.2).

### RNA extraction, library construction and sequencing

Total RNA was isolated from mulberry leaves after Mn stress treatments. These comprise the sufficiency (CK), deficiency (T0), moderate deficiency (T1), and toxicity (T2 and T3) treatments, using the Trizol reagent kit (Invitrogen, Carlsbad, CA, USA) according to the manufacturer’s instructions. The RNA quality was assessed on an Agilent 2100 Bioanalyzer (Agilent Technologies, Palo Alto, CA, USA) and checked using RNase-free agarose gel electrophoresis. mRNA was then enriched by Oligo (dT) beads after total RNA was extracted. The enriched mRNA was then fragmented into short fragments using fragmentation buffer and reverse transcribed into cDNA using the NEBNext Ultra RNA Library Prep Kit for Illumina (NEB #7530, New England Biolabs, Ipswich, MA, USA). The double-stranded cDNA fragments were end-repaired, base added and ligated to Illumina sequencing adapters after purification. AMPure XP Beads (1.0×) were then used to further purify the ligation reaction. Polymerase chain reaction (PCR) was then used to amplify the ligated product. Finally, the resulting cDNA library was sequenced on an Illumina Novaseq6000 platform by Gene Denovo Biotechnology Co. (Guangzhou, China). The raw sequencing data related to this work have been deposited in NCBI and can be accessed via the link and accession number below: (https://www.ncbi.nlm.nih.gov/search/all/?term=PRJNA1217365).

### Bioinformatics and data analysis of the RNA-seq results

Clean reads were obtained from the raw reads after RNA sequencing by filtering using fastp v0.18.0 [20] to remove reads containing adapters and reads containing more than 10% of unknown nucleotides (N) and to remove low-quality reads containing more than 50% of low quality (Q-value ≤ 20) bases. Further, the clean reads were mapped to the ribosomal RNA (rRNA) database using the short reads alignment tool bowtie2 (v2.2.8) [21]. The rRNA-mapped reads were removed, and the remaining clean reads were further used in assembly and gene abundance calculation. Furthermore, HISAT2 (v2.4) was used to map the obtained paired-end clean reads to the *M. notabilis* reference genome [22] with “-rna-strandness RF” and other parameters set as default. Gene abundance quantification was performed using StringTie v1.3.1 to assemble the mapped reads of each sample [23, 24] in a reference-based approach. RSEM (v1.3.3) [25] was used to calculate FPKM (fragments per kilobase of transcript per million mapped reads) of each transcription region to quantify the expression abundance and variations. Correlation and principal component (PCA) analysis of the samples was performed by R software v4.2. Differentially expressed genes (DEGs) analysis was performed by DESeq2 (v1.42.1) [26] and genes whose expressions met the criteria for the false discovery rate (FDR ≤ 0.05) and log2 fold change|log_2_FC|>=1, were regarded as differentially expressed genes/transcripts. Heatmap and volcano plots were performed on the DEGs using R packages to determine the direction and distribution patterns of the gene. Further, gene ontology (GO) (http://geneontology.org/) enrichment analysis was performed using FDR ≤ 0.05 as a threshold. GO terms with FDR ≤ 0.05 were defined as significantly enriched GO terms involving the DEGs. The KEGG pathways enrichment (Kyoto Encyclopedia of Genes and Genomes) analysis was performed using the KEGG database and the same criteria as the GO analysis was used. The *CAX3* gene was selected from the DEGs for functional validation because it was exclusively expressed and upregulated under Mn toxicity conditions. Additionally, *CAX3* has been reported to play a role in Mn transport and detoxification in plants. To confirm its function in Mn stress response in mulberry, we further characterized *CAX3* through gene silencing.

### Mulberry seedling culture and virus-induced gene silencing (VIGS)

The mulberry seeds were sown in nutrient soil and cultivated in a greenhouse at 28℃. After 4 weeks of culture, the seedlings with good growth and basically the same state were selected for the VIGS infection experiment. To silence the *MaCAX3* gene, the gene was first cloned using the polymerase chain reaction (PCR) technique following the study by Li et al. [14]. The primers used were designed using Snapgene (v6.0.2). Primers include *MaCAX3*-F: ATGGACGGCTCAAACGATC and *MaCAX3*-R: TCAAGCAGCCAAAGCTCCT. After cloning and recovery, the *MaCAX3* target gene was transferred into the PCC-TRV2-GFP carrier provided by NC Biotech (Hainan, China) to commence the gene silence experiment [14]. The primers used are as follows: *Macax3*-VIGS-F: AGTGGTCTCTGTCCAGTCCTggaagaacaccgcaaacca, *Macax3*-VIGS-R: GGTCTCAGCAGACCACAAGTtcctccaactgttggaccag, and GFP-F: ACCCTCGTGACCACCCTGAC, GFP-R: AGTTCACCTTGATGCCGTTC. After cloning and production of the recombinant plasmids of TRV2-GFP, TRV2-GFP-*MaCAX3* and blank (CK) were transferred into an Agrobacterium (GV3101). Samples of mulberry leaves with fluorescence were collected at 14, 16, 18, and 20 d after the Agrobacterium infection of the mulberry seedlings. The blank control group was the uninfected group, the no-load control group was the Agrobacterium infection group containing pTRV2-GFP plasmid, and the experimental group was the Agrobacterium infection group containing PTRV2-GFP-*MaCAX3* plasmid. After sampling of the leaves at 14, 16, 18, and 20 d after the infection, the relative expression levels of the *MaCAX3* gene in the three groups were detected by qRT-PCR. The primers were *MaCAX3*-F: GGCTCTCTTGCATTCACCATA and *MaCAX3*-R: GGATGGTTCAAGTTTAAGTGGTG and the internal reference gene was actin3 (HQ163775) with the primers: Actin-F: GCATGAAGATCAAGGTGGTG and Actin-R: CATCTGCTGGAAGGTGCTAA were used for the qRT-PCR using the cDNA from the samples from the 14, 16, 18 and 20 d as the templates. Three biological and technical replicates were set in each group, and the relative gene expression was calculated by 2^−ΔΔCt^ method [27], and the significance was verified by analysis of variance (ANOVA) using R statistical software v4.2 and significance was at *p* ≤ 0.05 using Tukey’s HSD.

### Heterologous expression of *MaCAX3* in yeast and growth curve under yeast converted with Mn treatment

The open reading frame (ORF) of *MaCAX3* and *Macax3*-*VIGS* was amplified with gene-specific primers inserted into the pYES2 yeast expression vector (Invitrogen, Carlsbad, CA, USA). The recombinant construct and the empty vector were separately transformed into the host strain BY4741 using the S. c. EasyComp™ Transformation Kit (Invitrogen, Carlsbad, CA, USA). Metal tolerance assays of the yeast transformants were conducted as described [14] with minor modifications. Briefly, the yeast transformants harboring the recombinant vector and empty vector were individually precultured in synthetic dextrose media without URA amino acid (SD-U liquid medium) until the OD_600_ value reached 1.0, then the yeast cells were diluted to10-fold (10^− 1^, 10^− 2^, 10^− 3^, 10^− 4^, 10^− 5^, respectively) with sterile water and cultured on SD-U induction plates for 3 d with Mn as 2 mM, 4 mM, and 8 mM Mn, or without Mn (0 mM). Each Mn concentration was treated with 3 repetitions. Colonies growth was then observed after on the medium containing the Mn and those without Mn.

To understand the Mn tolerance of *MaCAX3* yeast inverters, a single colony of transformed yeast BY4741 (containing an empty expression vector and target gene expression vector) was selected. The colonies were placed into a 5 mL SD-U liquid medium for activation culture and were incubated overnight at 220 rpm at 30℃. The OD_600_ value was measured to about 0.5. Afterwards, 1 mL of WT + PYES2, WT + *MaCAX3*, and WT + *Macax3*-VIGS bacterial solution were each cultured overnight. After the OD_600_ was adjusted to 0.5 and 0.1 ml, each solution was inoculated into 50 mL of SD-U liquid medium containing 4 mM Mn and those without Mn and the initial OD_600_ value was measured after being mixed evenly at 220 rpm at 30℃ for 60 h. Each bacterial solution was inoculated three times. The OD_600_ value was measured at an interval of 2–4 h, and the growth curve was drawn based on the OD_600_ value.

### Validation of the RNA-Seq results by qRT-PCR

To verify the DEGs obtained from RNA-Seq, we randomly selected 18 DEGs based on their involvement in the GO terms and KEGG pathways for the real-time quantitative polymerase chain reaction (qRT-PCR) validation. We followed the methods described in our previous study for the qRT-PCR analysis [28]. Mulberry leaf samples after Mn treatment used for the RNA-Seq analysis were also used for total RNA and cDNA synthesis for the qRT-PCR validation. Furthermore, fold changes in gene expression were estimated using the 2^−ΔΔCt^ method [27]. The genes and their primers used for the qRT-PCR validation are listed in Table S1.

## Results

### Determination of oxidation and antioxidant indicators

**Fig. 1:**
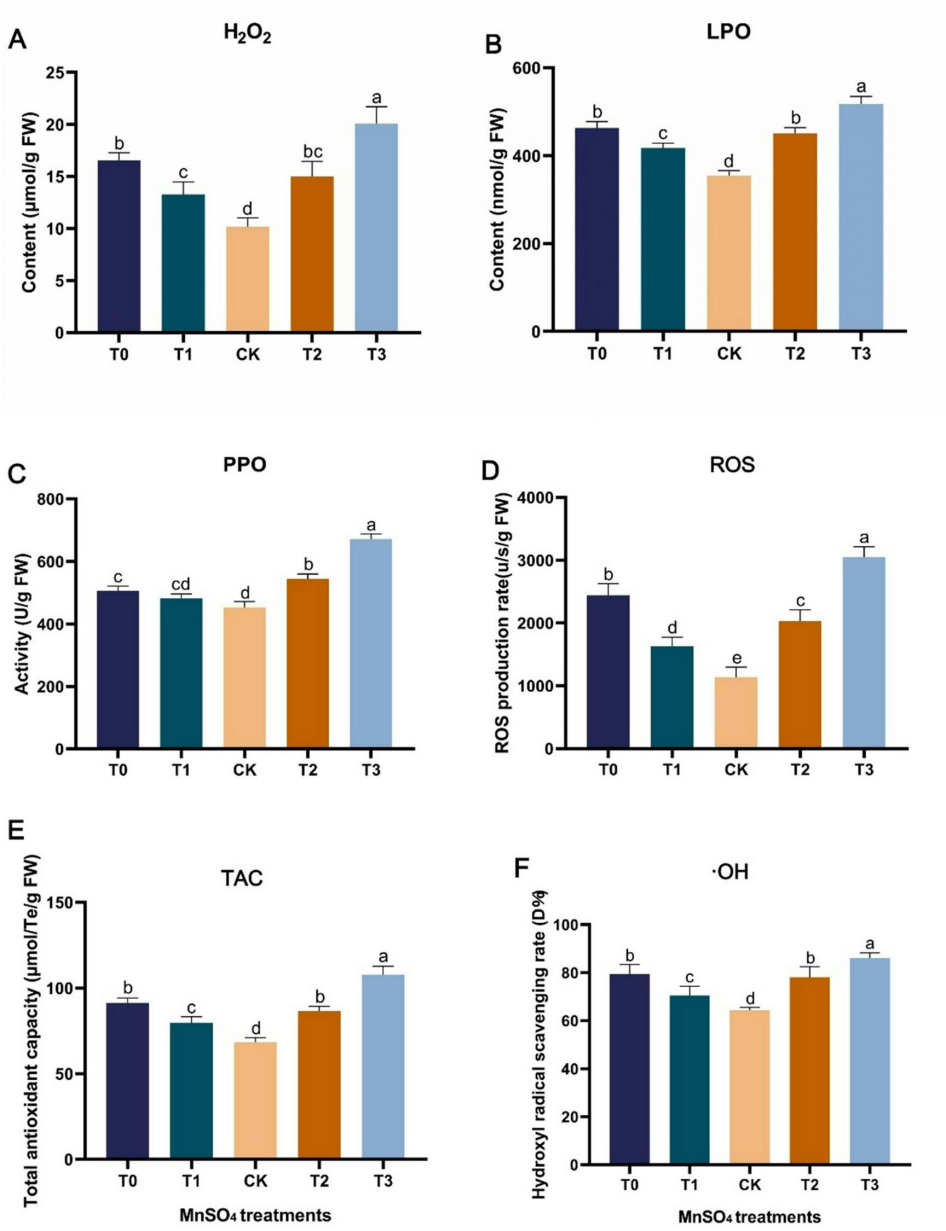
Effects of manganese stress on oxidation and antioxidant indicators of mulberry leaves. (**A**) Hydrogen peroxide. (**B**) Lipid peroxidase. (**C**) Polyphenol oxidase. (**D**) Reactive oxygen species. (**E**) Total antioxidant capacity. (**F**) Hydroxyl radical scavenging rate. T0: 0 mM MnSO_4_ treatment; T1: 0.03 mM MnSO_4_ treatment; CK: 0.15 mM MnSO_4_ treatment; T2: 1.5 mM MnSO_4_ treatment; T3: 3 mM MnSO_4_ treatment. Columns are values means of three replicates of leaf samples, and error bars represent the standard deviation of the three replicates. Different letters above the bars represent significant differences (Tukey’s HSD, *p* ≤ 0.05)

The results on growth, Mn mineral analysis in leaf, and physiological parameters have been previously reported in our study on Mn stress [1]. Oxidation and antioxidant parameters as impacted by Mn at various levels of concentration were evaluated in mulberry leaves (Fig. 1). Hydrogen peroxide (H_2_O_2_) and lipid peroxidase (LPO) contents were significantly elevated in the Mn-deficiency (T0), moderate deficiency (T1), moderate toxicity (T2) and toxicity (T3) groups as compared to the sufficiency group (CK) (Fig. 1A, B). Interestingly, H_2_O_2_ and LPO contents were significantly higher in the T0 and T3 compared to the CK. Polyphenol oxidase (PPO) activity increased in all the Mn treatment levels and most especially in the T2 and T3. The PPO activity levels in the T0, T2, and T3 were significantly different from CK (Fig. 1C). The reactive oxygen species (ROS) production rate was significantly higher in all treatment levels except for the CK (Fig. 1D), but the ROS production rate in T0 and T3 were the most prevalent. The total antioxidant capacity (TAC) level was also significantly increased in all the Mn deficiency and toxicity levels than in CK (Fig. 1E). A similar trend was observed in the hydroxyl radical (•OH) scavenging rate (Fig. 1F).

### Analysis of Mn stress of mulberry leaves on palisade tissue and spongy tissue structural, cell wall structure components and their related enzyme activities

**Fig. 2:**
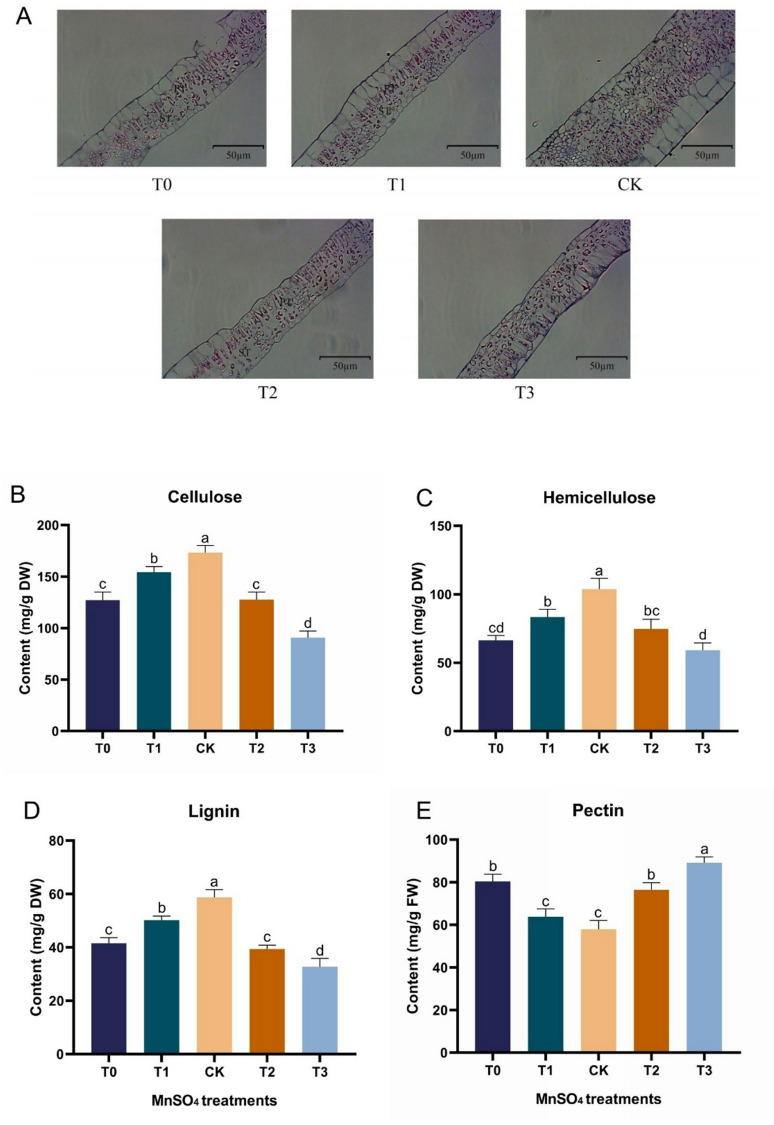
Effects of manganese stress on palisade tissue and spongy tissue structure and cell wall structural components of mulberry leaves. (**A**) Palisade tissue and spongy tissue structure; PT: palisade tissue. ST: spongy tissue. (**B**) Cellulose content. (**C**) Hemicellulose content. (**D**) Lignin content. (**E**) Pectin content. T0: 0 mM MnSO_4_ treatment; T1: 0.03 mM MnSO_4_ treatment; CK: 0.15 mM MnSO_4_ treatment; T2: 1.5 mM MnSO_4_ treatment; T3: 3 mM MnSO_4_ treatment. Columns are the mean values of three replicates of leaf samples, and error bars represent the standard deviation of the three replicates. Different letters above the bars represent significant differences (Tukey’s HSD, *p* ≤ 0.05)

As illustrated in Fig. 2A, the results reveal that the CK group (0.15 mM) displayed a tight arrangement of palisade tissue and minimal spongy tissue gaps, indicating a higher chlorophyll content, more effective light absorption, and higher water content. In the low Mn treatment (T1; 0.03 mM), both palisade and spongy tissues decreased, leading to an increasing disruption in the tissue structure and widening gaps, and even showing disorganized trends in the Mn-deficiency (T0; 0 mM). However, in the high Mn-toxicity treatments (T2; 1.5 mM and T3; 3 mM), the disruption increased with higher Mn concentrations in both palisade and spongy tissues, accompanied by a reduction in the thickness of the palisade tissue layer. Results of the cell wall structure components showed that the cellulose, hemicellulose, and lignin contents were significantly higher in the CK treatment compared to the T0-T3 (Fig. 2B-D). Nonetheless, the contents were higher in the Mn-deficiency groups (T0 and T1) compared to the Mn-toxicity groups (T2 and T3). The result further reveals that pectin content was higher under Mn-deficiency (T0) and Mn-toxicity conditions (T2, T3), with the highest level observed in T3. In contrast, the control group (CK) exhibited significantly lower pectin content (Fig. 2E). Cell wall structural components related to enzymes such as cellulase, xylanase, and pectinase were analyzed (Table 1). According to the results, cellulase activity was significantly lower in T2 (1067.35 µg /min /g) and T3 (957.69 µg /min /g), whereas the activity was relatively higher in the T0, T1, and the CK (Table 1). Likewise, the xylanase activity was higher in the T0-T3 compared to the CK (945.52 nmol/min/g) which was significantly (*p* ≤ 0.05) higher. Meanwhile, pectinase activity was lower in Mn-toxicity groups, T2 and T3, with T3 being the lowest (15.22 mg/h/g) compared to the Mn-deficiency (T0 and T1) and the CK (Table 1).

**Table 1:** Cell wall structure components enzyme activities

	Treatments				
Enzyme activity	T0	T1	CK	T2	T3
Cellulase (µg /min /g)	1147.79 ± 1.51^c^	1437.55 ± 0.67^a^	1357.87 ± 0.07^b^	1067.35 ± 1.75^c^	957.68 ± 1.18^d^
Xylanase (nmol/min/g)	1438.16 ± 0.96^b^	1044.50 ± 2.50^c^	945.52 ± 1.07^d^	1353.18 ± 2.92^b^	1680.89 ± 2.13^a^
Pectinase (mg/h/g)	18.21 ± 0.82^c^	20.47 ± 0.80^b^	23.60 ± 1.03^a^	17.14 ± 0.66^c^	15.22 ± 0.54^d^

Note: values are means of three replicates of leaf samples and standard deviation. Different letters (superscript) represent significant differences (Tukey’s HSD, *p* ≤ 0.05)

### Analysis of RNA-seq-based transcriptome profiles of mulberry response to Mn stresses

Mulberry leaves transcriptomic profiles, as altered by Mn-deficient and -toxic treatments, were investigated and analyzed via RNA-seq analysis. From the RNA-Seq analysis, 611,007,704 raw reads were obtained from the 15 libraries with an average of 40,733,846.93 raw reads per sample (Fig. S1A; Table S2). A total of 608,797,404 clean reads were obtained from the 15 libraries with an average of 40,586,493.6 per sample, representing 99.64% after filtering junctional sequencing contamination, adapters, and low-quality raw reads (Fig.S1B; Table S2). After base composition analysis of the clean reads, the GC content of the sample reads (on average) was 46.52%, and Q20 and Q30 scores were 97.69% and 93.41%, respectively (Table S2). After mapping the clean reads to the *M. notabilis* reference genome, approximately 75.56% (average) of the clean reads were uniquely mapped, with only 22% unmapped, and 78% of the clean reads were mapped to the reference genome (Table S2). Mapping of the clean reads to the reference genome showed that most of the clean reads were mapped mostly to the exon region (88.63%; on average) (Fig. S1C; Table S2). The FPKM distribution of the gene and the density of gene expression (even distribution of reads) in all the samples are shown in Fig. S1D, E. Principal component (PC) analysis shows a clear separation of the sample’s treatment groups, and PC1 and PC2 reveal 45.9 and 28.6% variation, respectively (Fig. S1F). Pearson’s correlation heatmap of the samples indicated that the correlation coefficient (R^2^) was more than 0.9, indicating the reliability of the data samples (Fig. S1G). The profiling results showed 174 (CK), 74 (T0), 79 (T1), 72 (T2), and 46 (T3) genes were uniquely expressed in the individual levels whereas 14,284 genes were profiled amongst all the samples treated (CK, T0-T3) and many genes were expressed within and between treatments (Fig. S1H).

### Analysis of differentially expressed genes (DEGs) in mulberry response to Mn deficiency and toxicity

**Fig. 3:**
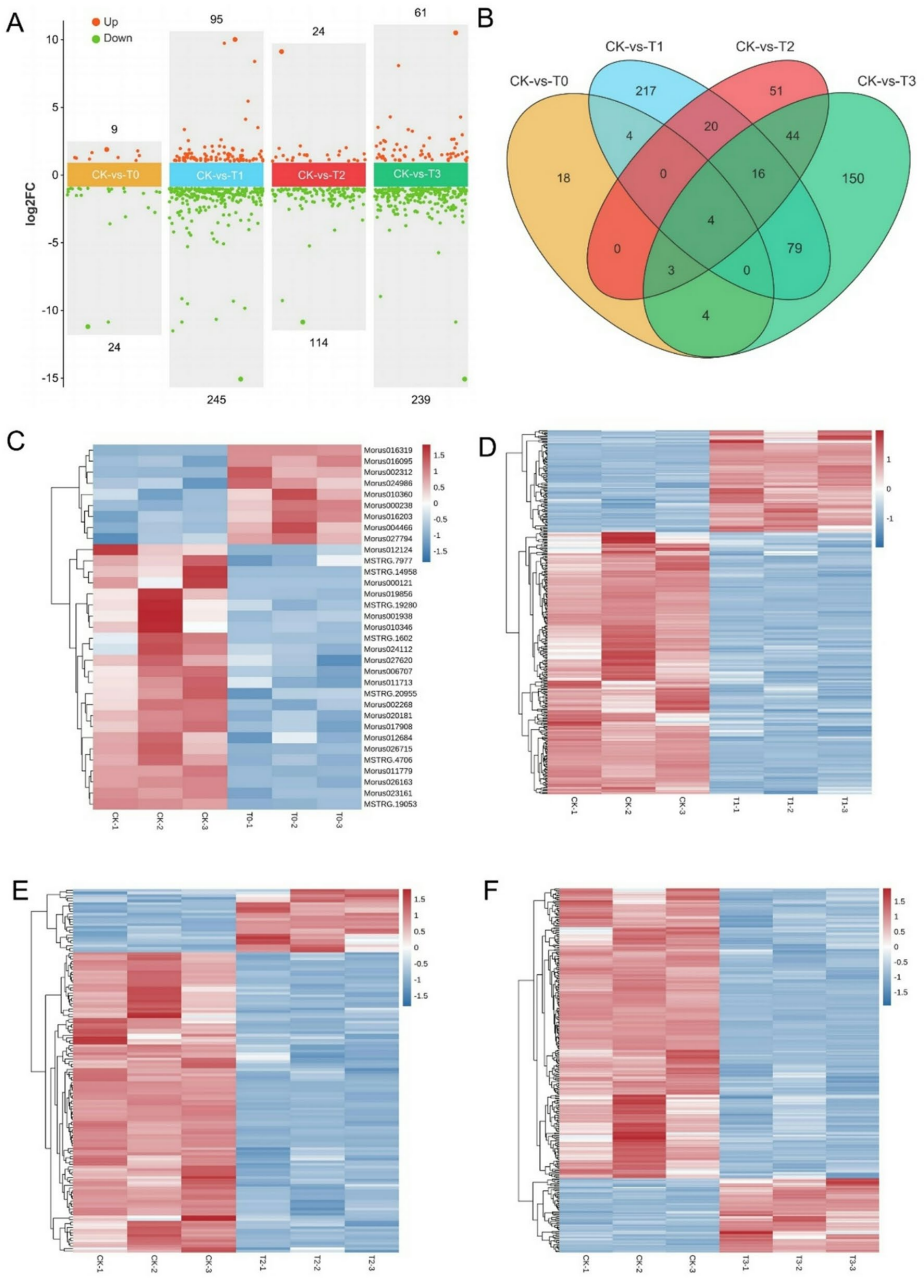
Differentially expressed genes (DEGs) statistics. (**A**) DEGs statistics and expression patterns. (**B**) Venn diagram showing the DEGs in mulberry plants exposed to Mn deficiency (CK-vs-T0), moderate deficiency (CK-vs-T1), moderate toxicity (CK-vs-T2), and toxicity (T3). (**C**-**F**) Heatmap showing the DEGs patterns in CK-vs-T0, CK-vs-T1, CK-vs-T2 and CK-vs-T3, respectively. Red and blue colors in the heatmap represent the upregulation and downregulation of the genes

Analysis of gene expressions in mulberry’s response to Mn-deficiency and toxicity reveals that a total of 28,394 unigenes were obtained and 811 of them were DEGs in CK treatments comparisons with the T0-T3, representing 2.86% of the total genes expressed. Among the 811 DEGs, 189 were upregulated and 622 were downregulated. In the Mn-deficiency groups, 33 DEGs (9-up and 24-downregulated) were obtained in the CK-vs-T0 whereas CK-vs-T1 revealed 340 DEGS comprising 95 upregulated and 245 downregulated genes (Fig. 3A). In the toxicity groups, 138 DEGs consisting of 24 upregulated and 114 downregulation were in the CK-vs-T2 whereas 300 DEGs including 61 upregulated and 239 downregulation in the CK-vs-T3 were obtained (Fig. 3A**).** The result showed that the moderate Mn-deficiency (CK-vs-T1) and high Mn-toxicity (CK-vs-T3) exhibited more DEGs but Mn-deficiency (CK-vs-T0; that is no Mn supply) exhibited very low DEGs in mulberry. Further, Venn diagram analysis indicates that 18, 217, 51, and 150 DEGs were uniquely expressed in CK-vs-T0, CK-vs-T1, CK-vs-T2, and CK-vs-T3, respectively. However, 4 DEGs were commonly expressed among all the treatment groups (Fig. 3B). Volcano plots (Fig. S2) and cluster heatmap analysis (Fig. 3C-F) reveal the contrasting pattern of the DEGs in each treatment group comparison with the control.

### Gene ontology (GO) annotation and enrichment analysis of the DEGs

The gene ontology (GO) involving the DEGs was analyzed and presented by heatmap. The results indicate that the DEGs were implicated in three GO categories including biological process (BP), cellular component (CC), and molecular function (MF) with the BP category being the dominant (Fig. S3). Based on p-value (*p* ≤ 0.05), 21 GO terms including cytoplasmic microtubule depolymerization (GO:0010938), negative regulation of mRNA processing (GO:0050686 ), cellular homeostasis (GO:0019725), etc. involving the DEGs were significantly implicated in the CK-vs-T0 (Fig. S3A). In the CK-vs-T1, 40 GO terms involving the DEGs were significantly implicated, and these include polysaccharide catabolic process (GO:0000272), DNA replication proofreading (GO:0045004), L-asparagine biosynthetic process (GO:0070981) and others (Fig. S3A). Meanwhile, in the CK-vs-T2, 27 GO terms were significantly involved with the DEGs. These included plant-type cell wall organization or biogenesis (GO:0071669), abscisic acid catabolic process (GO:0046345), cell wall modification (GO:0042545), lipid catabolic process (GO:0016042), superoxide metabolic process (GO:0006801), response to reactive oxygen species (GO:0000302), and others (Fig. S3A). On the side of the CK-vs-T3, 50 GO terms were significant with the DEGs and among them included protein refolding (GO:0042026), photosynthetic electron transport chain (GO:0009767), transmembrane transport (GO:0055085), inorganic ion transmembrane transport (GO:0098660), etc. (Fig. S3A). Based on based on FDR-value (FDR value ≤ 0.05), 6, 21, 14, and 19 GO terms were significant with the DEGs in CK-vs-T0, CK-vs-T1, CK-vs-T2, and CK-vs-T3, respectively (Fig. S3B).

**Fig. 4:**
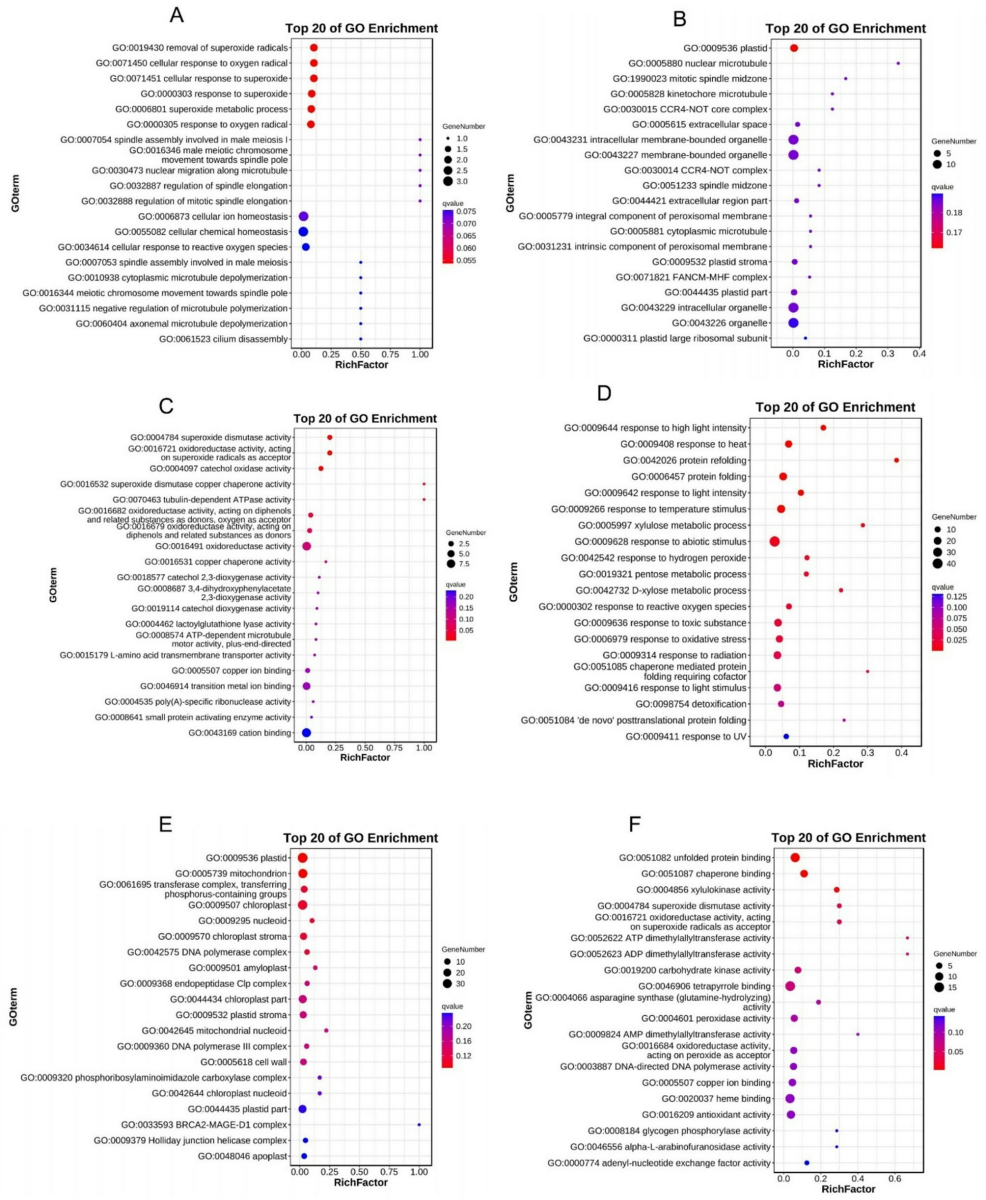
Top 20 gene ontology (GO) functional annotation enrichment analysis of the DEGs. (**A**-**C**) GO enrichment in biological process, cellular component, and molecular function respectively in CK-vs-T0. (**D**-**F**) GO enrichment in biological process, cellular component, and molecular function respectively in CK-vs-T1. The colors represent the concentration of DEGs. The bubble size represents the number of DEGs in the GO terms

**Fig. 5:**
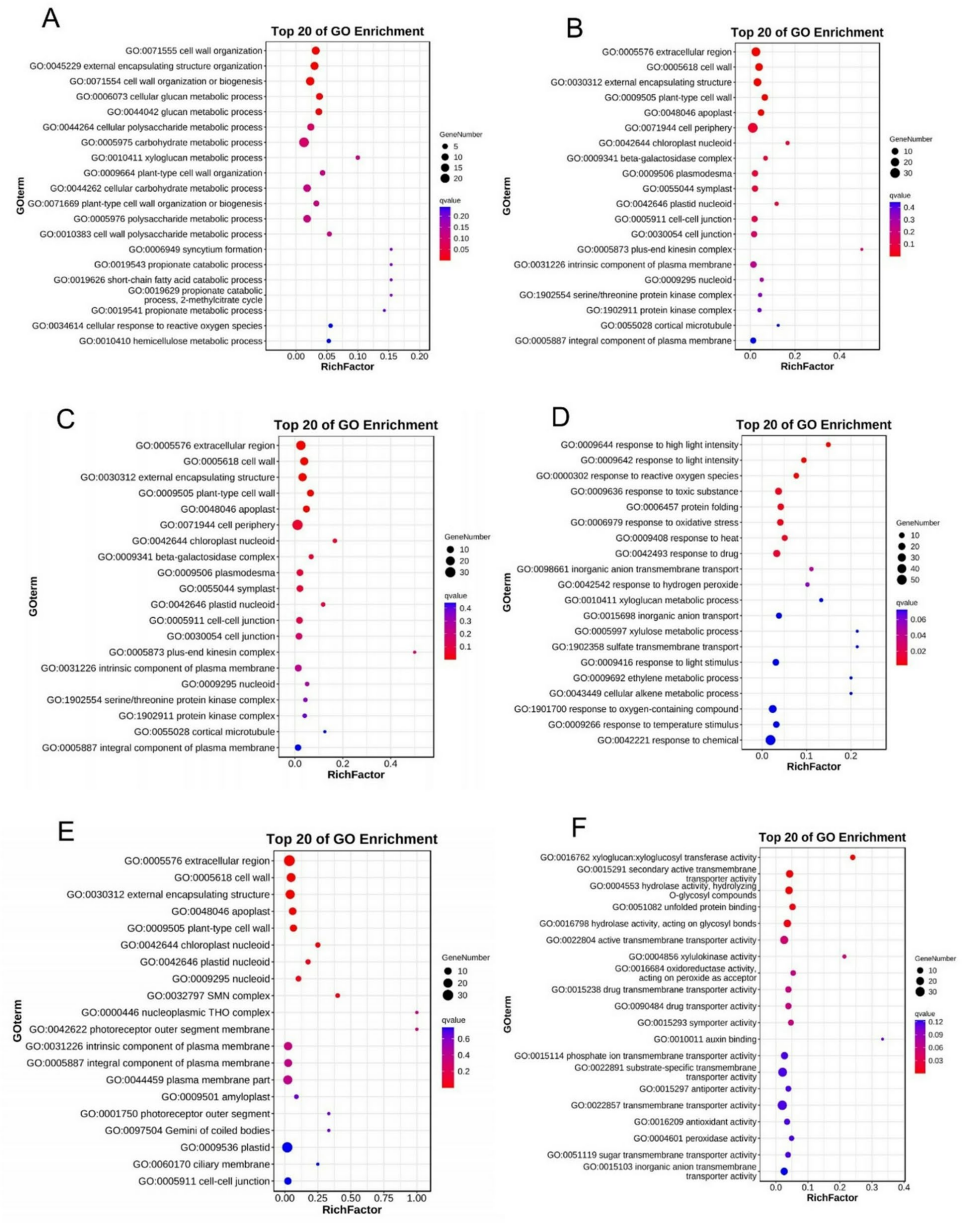
Top 20 of gene ontology (GO) functional annotation enrichment analysis of the DEGs. (**A**-**C**) GO enrichment in biological process, cellular component, and molecular function respectively in CK-vs-T2. (**D**-**F**) GO enrichment in biological process, cellular component, and molecular function respectively in CK-vs-T3. The colors represent the concentration of DEGs. The bubble size represents the number of DEGs in the GO terms

Analysis of the top 20 GO enrichments of the DEGs reveal that in the CK-vs-T0, the GO terms such as removal of superoxide radicals (GO:0019430), response to oxygen radical (GO:0000305), superoxide metabolic process (GO:0006801), cellular response to superoxide (GO:0071451), etc., were significantly enriched in the biological process (BP) (Fig. 4A), whereas plastid (GO:0009536), intracellular membrane-bounded organelle (GO:0043231), membrane-bounded organelle (GO:0043227), and others were enriched in the cellular component (CC) (Fig. 4B) and superoxide dismutase activity (GO:0004784), oxidoreductase activity, acting on superoxide radicals as acceptor (GO:0016721), superoxide dismutase copper chaperone activity (GO:0016532), tubulin-dependent ATPase activity (GO:0070463), etc., were enriched in the molecular function (MF) (Fig. 4C). In the CK-vs-T1, GO terms including protein refolding (GO:0042026), protein folding (GO:0006457), response to hydrogen peroxide (GO:0042542), response to reactive oxygen species (GO:0000302), response to toxic substance (GO:0009636), response to oxidative stress (GO:0006979), detoxification (GO:0098754), etc., were enriched in the BP (Fig. 4D). Meanwhile, in the CC, plastid, mitochondrion (GO:0005739), chloroplast (GO:0009507), cell wall (GO:0005618), etc., were enriched (Fig. 4E), however, unfolded protein binding (GO:0051082), chaperone binding (GO:0051087), xylulokinase activity (GO:0004856), etc., were among the assigned GO terms enriched in the MF category (Fig. 4F). Again, in the CK-vs-T2 (Fig. 5A), cell wall organization (GO:0071555), cellular glucan metabolic process (GO:0006073), carbohydrate metabolic process (GO:0005975) are among the most enriched GO terms in the BP. Also, plant-type cell wall (GO:0009505), cell periphery (GO:0071944), cell wall and many others were the most enriched GO terms in the CC (Fig. 5B), whereas GO terms in the MF follows a similar trend (Fig. 5C). In the CK-vs-T3, a similar pattern regarding the GO terms enrichment as seen in the CK-vs-T2 was observed (Fig. 5D-F).

**Fig. 6:**
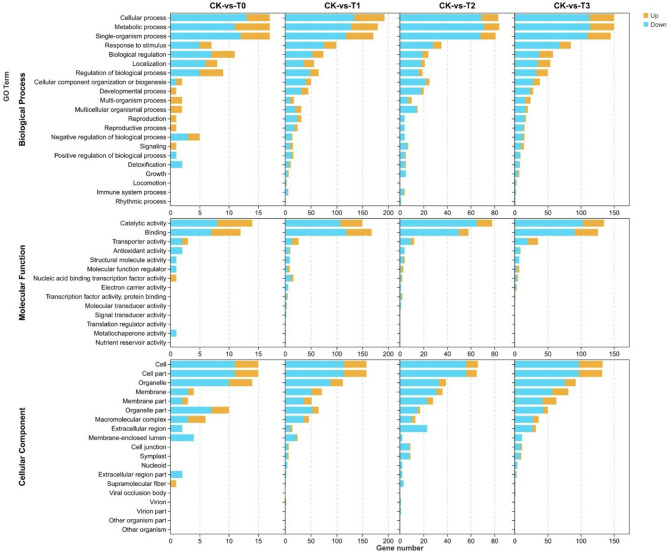
Secondary classification of gene ontology (GO) functional annotation analysis of the DEGs. The yellow-gold color represents upregulated genes, and the blue color represents downregulated genes. The length of the bar shows the gene number in the GO terms

We further analyzed the secondary GO terms involving the DEGs in each GO category. From the results, the DEGs were involved in detoxification, growth, signaling, cellular process, metabolic process, etc., in the BP category (Fig. 6). In the MF category, the DEGs were mainly involved in the GO secondary terms of catalytic activity, binding, antioxidants, transporter activity, and many others. Meanwhile, in the CC, the DEGs were mostly involved in the cell, cell part, membrane, organelle part, membrane part, etc., (Fig. 6).

### KEGG annotation and enrichment analysis of the DEGs

**Fig. 7:**
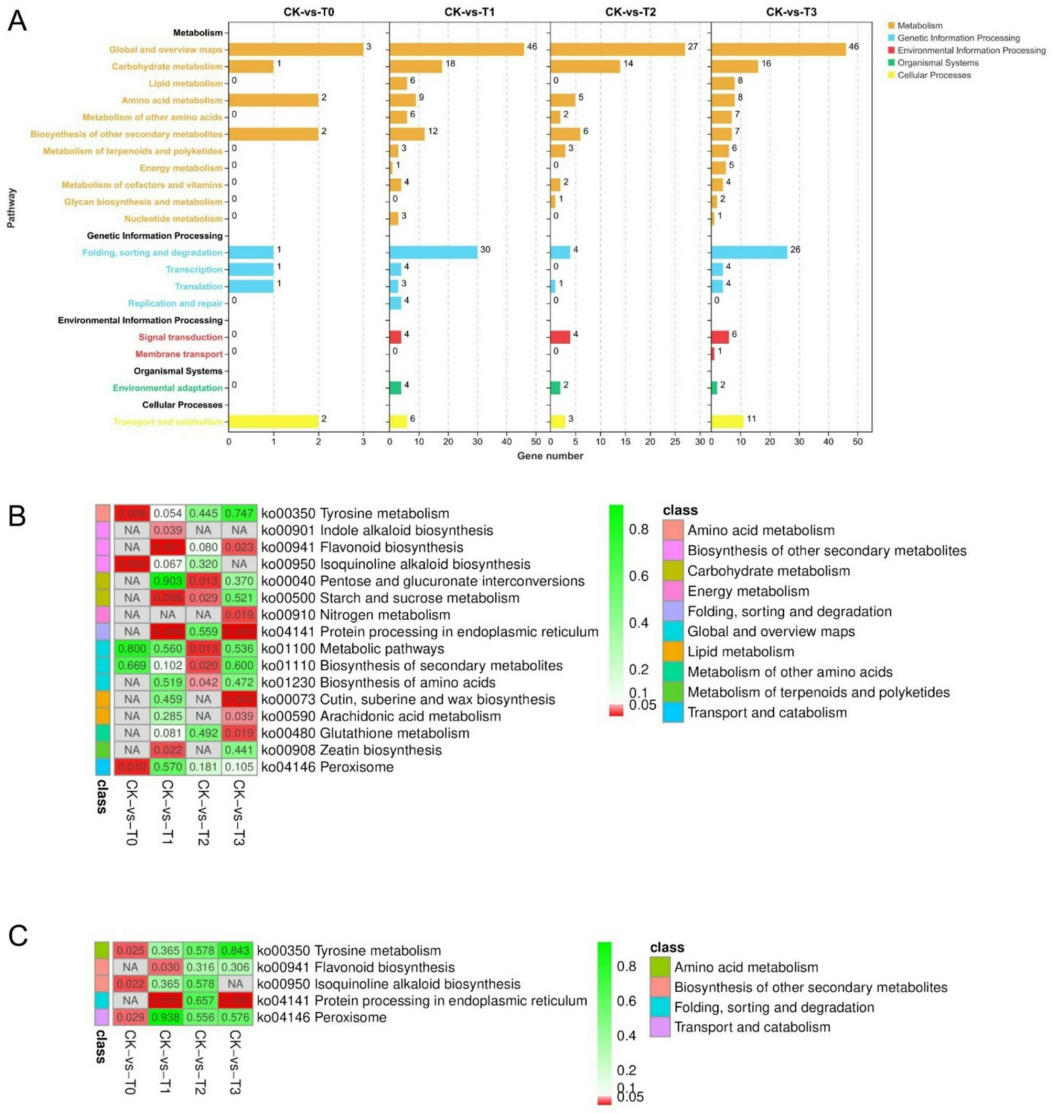
KEGG annotation and classification involving the DEGs analysis. (**A**) KEGG-pathway annotation. Heatmap of KEGG pathway classification analysis based on p-value (**B**) and FDR (**C**)

**Fig. 8:**
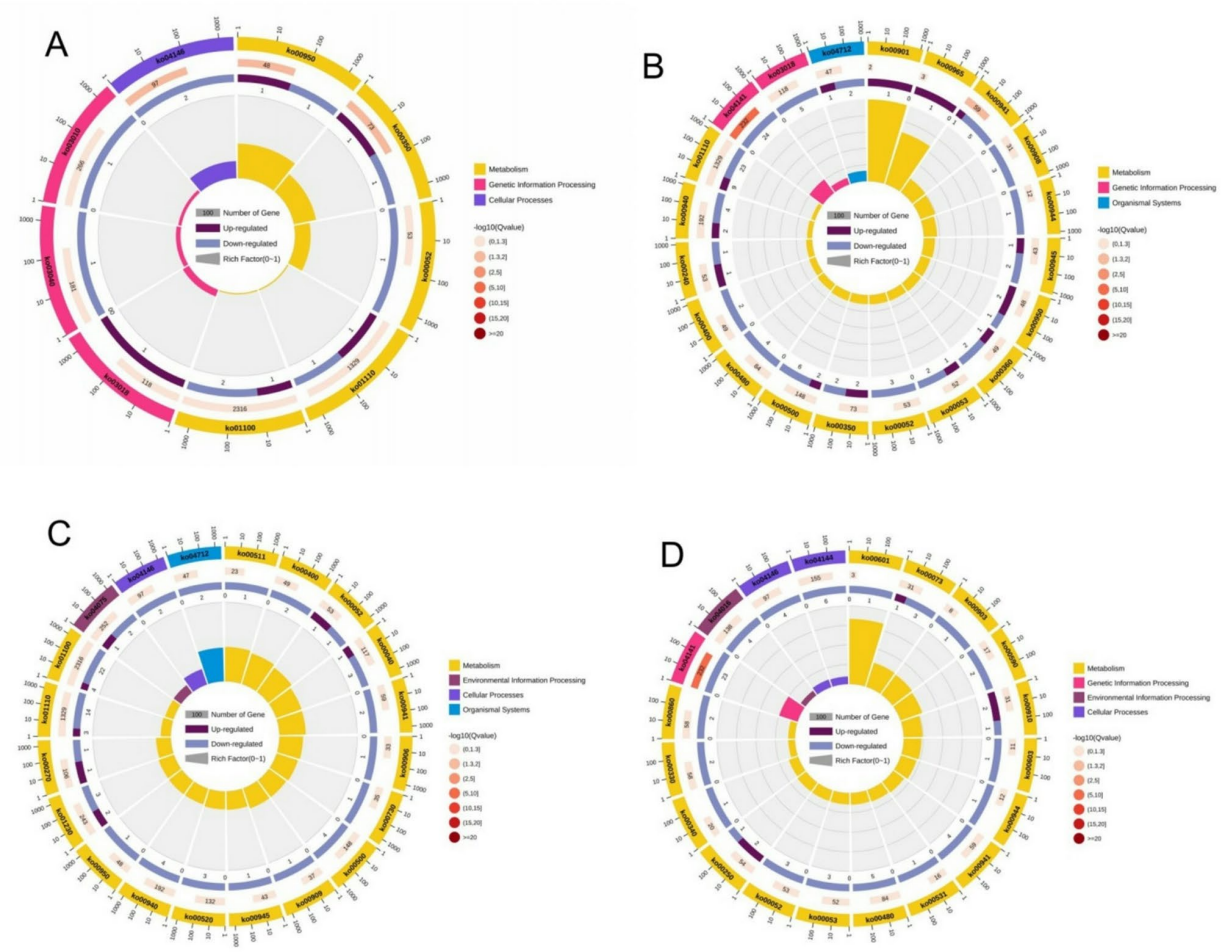
KEGG enrichment involving the DEGs analysis. (**A**-**D**) KEGG-pathway enrichment in CK-vs-T0, CK-vs-T1, CK-vs-T2, CK-vs-T3, respectively. From the outer circle to the inner circle: Circle 1: The top 20 enriched class A KEGG pathways of DEGs, and the coordinate scale outside the circle is the number of DEGs. Yellow represents the KEGG pathway of metabolism. Circle 2: The background of DEGs enriched in each pathway. The greater the number of DEGs, the longer the bar, and the smaller the q-value, the redder the color. Circle 3: The bar of the proportion of up-regulated (dark purple) and down-regulated (light purple) DEGs enriched in each pathway. The specific values are shown below. Circle 4: The rich factor value of each pathway (the number of DEGs divided by the total number of genes in the pathway); each grid line of the background grid represents 0.1

Analysis of the pathways enriched with the DEGs was performed using the Kyoto Encyclopedia of Genes and Genomes (KEGG) database. From the results, the DEGs were assigned to 5 main KEGG classes including metabolism (11 sub-class), genetic information processing (4 sub-class), environmental information processing (2 sub-class), organismal systems, and cellular process (1-sub-class each) (Fig. 7A). Further analysis of the pathways of the DEGs under these classes reveals that in the CK-vs-T0, only three pathways such as peroxisome (ko04146), tyrosine metabolism (ko00350), and isoquinoline alkaloid biosynthesis (ko00950) were significant (Fig. 7B). Meanwhile, pathways such as zeatin biosynthesis (ko00908), protein processing in endoplasmic reticulum (ko04141), starch and sucrose metabolism (ko00500), flavonoid biosynthesis (ko00941) and indole alkaloid biosynthesis (ko00901) were significant in the CK-vs-T1. Furthermore, 5 significant pathways including pentose and glucuronate interconversions (ko00040), biosynthesis of secondary metabolites (ko01110), biosynthesis of amino acids (ko01230), etc., were significant in CK-vs-T2. On the other hand, glutathione metabolism (ko00480), arachidonic acid metabolism (ko00590), etc., were significant in CK-vs-T3 (Fig. 7B). Analysis of the pathways based on FDR-value (FDR value ≤ 0.05) shows only 5 pathways namely, peroxisome, protein processing in endoplasmic reticulum, isoquinoline alkaloid biosynthesis, flavonoid biosynthesis, and tyrosine metabolism were significant in either the CK-vs-T0, CK-vs-T1, CK-vs-T2 or CK-vs-T3 (Fig. 7C**)**. The enrichment analysis of the DEGs shows that the genes were enriched mainly in metabolism and genetic information processing (Fig. 8A-D).

### Analysis of DEGs related to oxidation, antioxidant, and detoxification

**Fig. 9:**
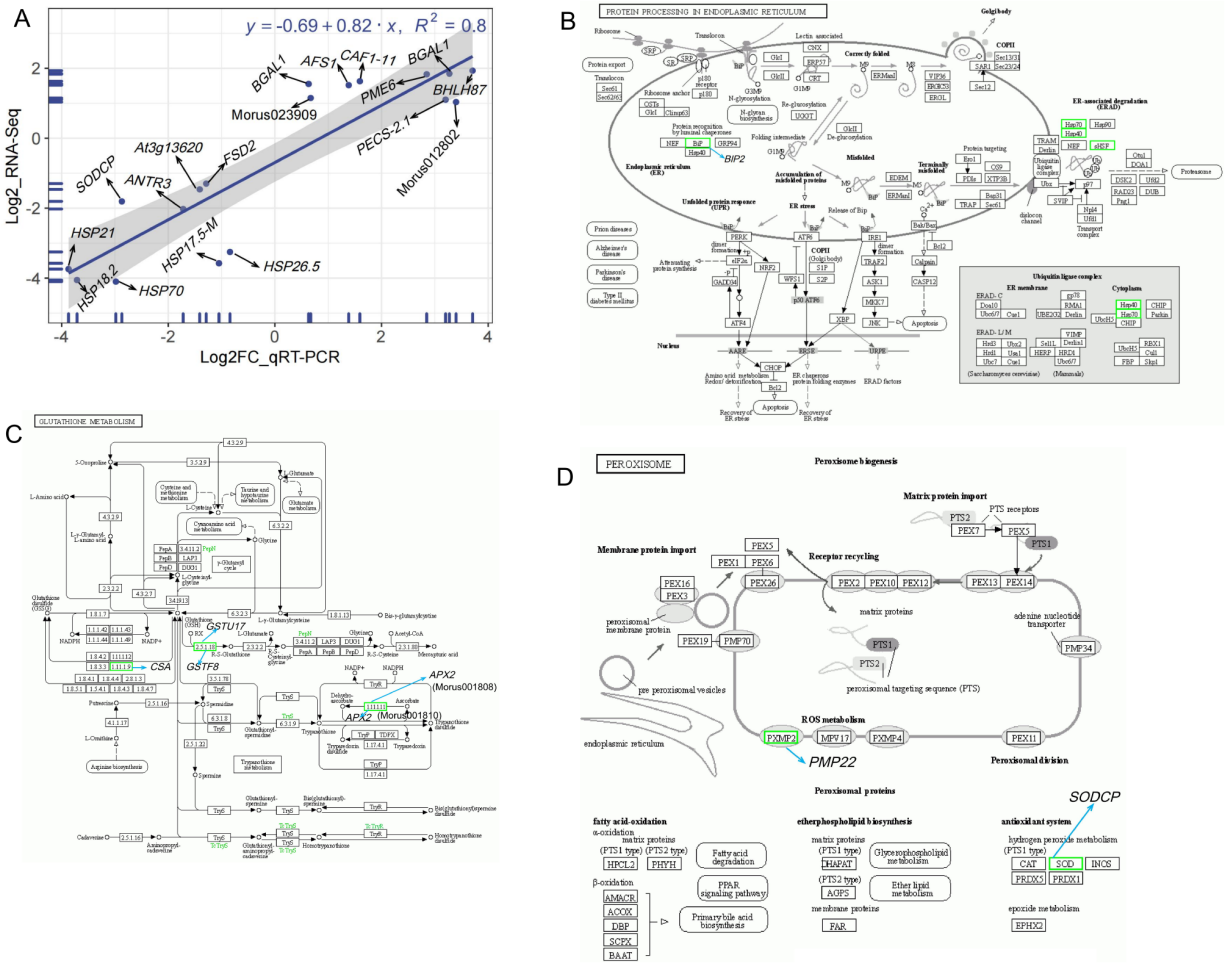
(**A**) Regression line plot showing quantitative real-time polymerase chain reaction (qRT-PCR) for the relative expression level and RNA sequencing (RNA-seq) results of the differentially expressed genes (DEGs). The mulberry actin3 (HQ163775.1) gene was used as the reference gene. All the samples were conducted with three biological and technical replicates. The relative expression levels were calculated as 2^−∆∆CT^. The y-axis represents the log2FC of RNA-seq, and the x-axis denotes the log2 fold change in relative expression obtained by qRT-PCR. Molecular mechanisms of significant DEGs in KEGG pathways involved in protein processing (**B**) glutathione metabolism (**C**) Peroxisome (**D**). Genes in green fonts represent downregulation

A total of 5 DEGs in CK-vs-T0, 10 DEGs in CK-vs-T1, 4 DEGs in Ck-vs-T2, and 9 DEGs in CK-vs-T3 were related to antioxidants. Also, genes such as *CCS* (copper chaperone for superoxide dismutase), *SODCP* (sodC protein), and *PPO* (Morus012124; polyphenol oxidase) were all downregulated but *PPO* (Morus002312; polyphenol oxidase) was upregulated in CK-vs-T0 (Table 2). In the CK-vs-T1, genes including *SODCP*, *APX2*, *GSTF8* (glutathione S-transferase) (L-ascorbate peroxidase 2), etc., were downregulated whereas *DOX2* (Prostaglandin G/H synthase 1), *PER21* (peroxidase 21) were upregulated (Fig. 9). In the toxicity groups, *FSD3* (protein Fe superoxide dismutase 1), *FSD2* (superoxide dismutase [Fe], chloroplastic), etc., were downregulated in CK-vs-T2 and CK-vs-T3, however, *PNC2* (cationic peroxidase 2) was downregulated and only altered in CK-vs-T2 (Table 2). Interestingly, *GLT1* (Glutamate synthase [NADH] was upregulated and only altered in CK-vs-T3. As shown in Table 2, most of the genes were also related to detoxification. Among them, *GSTU17* (glutathione S-transferase U17), *GSTF8* (glutathione S-transferase), and *CAD* (probable mannitol dehydrogenase) were downregulated in the Mn-treated plants. Meanwhile, *CCS* and *SODCP* were only altered in Mn-deficiency (CK-vs-T0) plants. However, *CAX3* (Vacuolar cation/proton exchanger 3) was upregulated in the Mn-toxicity groups and *HIPP09* (heavy metal-associated isoprenylated plant protein 9) was upregulated only in CK-vs-T1. Genes such as *CYP71AN24* (cytochrome P450 71A1), *LSC30* (Ferritin-3), *ACO1*(1-aminocyclopropane-1-carboxylate oxidase 1) were among the many genes related to oxidation and were upregulated in either CK-vs-T0 and the Mn-treated groups (Fig. 9; Table 2).

**Table 2:** Differentially expressed genes (DEGs) related to antioxidants, detoxification, and oxidation

		CK-vs-T0	CK-vs-T1	CK-vs-T2	CK-vs-T3	
	Antioxidants related genes					
ID	Symbol	log_2_(fc)	log_2_(fc)	log_2_(fc)	log_2_(fc)	Description
Morus023161	*CCS*	-1.01	-1.53			Copper chaperone for superoxide dismutase
Morus011779	*SODCP*	-1.54	-1.81			sodC protein
Morus002312	*PPO*	1.41				Polyphenol oxidase
Morus012124	*PPO*	-3.63				Polyphenol oxidase
Morus017399	*DOX2*		1.34			Prostaglandin G/H synthase 1
Morus024421	*PER21*		2.10			peroxidase 21
Morus001808	*APX2*		-3.95		-2.05	L-ascorbate peroxidase 2
Morus001810	*APX2*		-2.80		-2.42	L-ascorbate peroxidase 2
Morus008867	*PER19*		-1.22	-1.59	-2.56	peroxidase 19
Morus011651	*GSTF8*		-1.10		-1.31	glutathione S-transferase
Morus012342	*PCMP-E94*		-1.49			hypothetical protein L484_012342
Morus023118	*FSD3*		-1.38	-1.52	-1.56	Protein Fe superoxide dismutase 1
Morus017226	*PNC2*			-5.26		cationic peroxidase 2
Morus017091	*FSD2*			-1.46	-1.22	superoxide dismutase [Fe], chloroplastic
Morus006835	*GLT1*				1.07	Glutamate synthase [NADH]
Morus013081	*PER47*				-1.63	Peroxidase 47
Morus024292	*CSA*				-1.20	probable phospholipid hydroperoxide glutathione peroxidase
	Detoxification related genes					
Morus023161	*CCS*	-1.01	-1.53			Copper chaperone for superoxide dismutase
Morus011779	*SODCP*	-1.54	-1.81			sodC protein
Morus002603	*HIPP09*		1.15			heavy metal-associated isoprenylated plant protein 9
Morus017399	*DOX2*		1.34			Prostaglandin G/H synthase 1
Morus024421	*PER21*		2.10			peroxidase 21
Morus002334	*CAD*		-2.62		-1.51	probable mannitol dehydrogenase
Morus008867	*PER19*		-1.22	-1.59	-2.56	peroxidase 19
Morus011651	*GSTF8*		-1.10		-1.31	glutathione S-transferase
Morus021055	*GSTU17*		-1.66	-1.15	-1.08	glutathione S-transferase U17
Morus023118	*FSD3*		-1.38	-1.52	-1.56	Protein Fe superoxide dismutase 1
Morus026731	*CAD*		-1.25			probable mannitol dehydrogenase
Morus022009	*CAX3*			2.12	3.21	Vacuolar cation/proton exchanger 3
Morus017091	*FSD2*			-1.46	-1.22	superoxide dismutase [Fe], chloroplastic
Morus013081	*PER47*				-1.63	Peroxidase 47
	Oxidation related genes					
Morus023161	*CCS*	-1.01	-1.53			Copper chaperone for superoxide dismutase
Morus011779	*SODCP*	-1.54	-1.81			sodC protein
Morus002312	*PPO*	1.41				Polyphenol oxidase
Morus012124	*PPO*	-3.63				Polyphenol oxidase
Morus00446	*CYP71AN24*	1.27				cytochrome P450 71A1
Morus012684	*CYP78A5*	-1.49				cytochrome P450 78A5
Morus017908	*KAB1*	-1.26				probable voltage-gated potassium channel subunit beta
Morus002575	*HSP21*		-3.74		-2.59	small heat shock protein, chloroplastic
Morus008243	*LSC30*		1.58			Ferritin-3
Morus012384	*HSP70-8*		-2.92		-2.03	heat shock 70 kDa protein 8
Morus018780	*GOLS1*		-4.30		-2.73	galactinol synthase 1
Morus022488	*BAG6*		-2.01		-1.55	BAG family molecular chaperone regulator 6
Morus025782	*HSP26.5*		-4.75		-3.25	hypothetical protein L484_025782
Morus023118	*FSD3*		-1.38		-1.56	Protein Fe superoxide dismutase 1
Morus012384	*HSP70-8*		-2.92			heat shock 70 kDa protein 8
Morus001808	*APX2*		-3.95		-2.05	L-ascorbate peroxidase 2
Morus001810	*APX2*		-2.80		-2.42	L-ascorbate peroxidase 2
Morus024421	*PER21*		2.10			peroxidase 21
Morus008867	*PER19*		-1.22		-2.56	peroxidase 19
Morus017399	*DOX2*		1.34			Prostaglandin G/H synthase 1
Morus016963	*RE*			-1.01		protein RETICULATA, chloroplastic
Morus023118	*FSD3*		-1.38	-1.52		Protein Fe superoxide dismutase 1
Morus017091	*FSD2*			-1.46	-1.22	superoxide dismutase [Fe], chloroplastic
Morus027261	*ACO1*			1.41	1.35	1-aminocyclopropane-1-carboxylate oxidase 1
Morus005024	*HSP15.7*				-1.18	15.7 kDa heat shock protein, peroxisomal
Morus024218	*ACS1*				-1.62	1-aminocyclopropane-1-carboxylate synthase
Morus024292	*CSA*				-1.20	probable phospholipid hydroperoxide glutathione peroxidase

### Analysis of DEGs related to transporters, catabolism, and protein processing

Analysis of the DEGs reveals that several genes related to transporters were altered (Table 3). Five genes such as *SODCP* (sodC protein), *PMP22* (Peroxisomal membrane protein), and *At3g13620* (Serine/threonine exchanger SteT) were altered and downregulated but *PUB18* (U-box domain-containing protein 18 was upregulated in the CK-vs-T0 (Fig. 9D). Mn-treated plants exhibited a lot of altered transporters genes. In the CK-vs-T1, 23 DEGs of transporters (11 up and 12 downregulated) were altered. In the CK-vs-T2, 11 DEGs (2 up and 9 downregulated) were altered whereas 28 DEGs of transporters (11 upregulated and 17 downregulated) were altered in CK-vs-T2 (Table 3). *SWEET16* (Bidirectional sugar transporter), *KING1* (kinase regulatory subunit gamma-1), *TED6* (proline-rich receptor-like protein kinase PERK2), *NPF4.3* (protein NRT1/ PTR FAMILY 4.3), *DTX49* (protein DETOXIFICATION 49), *ABCG8* (ABC transporter G family member 8) etc., were altered and upregulated only in CK-vs-T1. Meanwhile, in the CK-vs-T2, *CAX3* (Vacuolar cation/proton exchanger 3) and *At3g13620* were the only upregulated transporter genes. *CAX3*,* At3g13620*,* BOR4* (boron transporter 4), *POR1* (mitochondrial outer membrane protein porin of 36 kDa), *CCX1* (cation/calcium exchanger 1), *PLT6* (probable polyol transporter 6), *ABCG6* (ABC transporter G family member 16), *ABCB11* (ABC transporter B family member 11), etc., were upregulated in CK-vs-T3 (Table 3).

**Table 3:** Differentially expressed genes related to transporters and catabolism

		CK-vs-T0	CK-vs-T1	CK-vs-T2	CK-vs-T3	
ID	Symbol	log_2_(fc)	log_2_(fc)	log_2_(fc)	log_2_(fc)	Description
Morus027794	*PUB18*	1.86				U-box domain-containing protein 18
Morus000121		-11.23				hypothetical protein L484_000121
Morus002268	*At3g13620*	-1.30		1.10	2.11	Serine/threonine exchanger SteT
Morus022009	*CAX3*			2.12	3.21	Vacuolar cation/proton exchanger 3
Morus011713	*PMP22*	-1.38				Peroxisomal membrane protein
Morus011779	*SODCP*	-1.54				sodC protein
Morus000972	*LAC7*		9.71			hypothetical protein L484_000972
Morus003052	*SWEET16*		1.14			Bidirectional sugar transporter
Morus005504	*KING1*		1.09			SNF1-related protein kinase regulatory subunit gamma-1
Morus007306	*TED6*		1.15			proline-rich receptor-like protein kinase PERK2
Morus009110	*SULTR3;1*		1.05			sulfate transporter 3.1
Morus026165	*SULTR3;4*			-2.31	-2.33	putative sulfate transporter 3.4
Morus016614	*SULTR2;1*		-1.03		1.27	Sulfate transporter 2.1
Morus012299	*NPF5.1*				-1.52	protein NRT1/ PTR FAMILY 5.1
Morus010975	*NPF4.3*		1.49		1.40	protein NRT1/ PTR FAMILY 4.3
Morus015735	*DTX49*		1.10			protein DETOXIFICATION 49
Morus027104	*DTX14*				-1.82	protein DETOXIFICATION 14
Morus019236	*OCT3*		1.07			organic cation/carnitine transporter 3
Morus023639	*LAC7*		5.43		4.27	laccase-7
Morus023963	*NPF5.4*		1.30			protein NRT1/ PTR FAMILY 5.4
Morus003585	*HKT1*		-1.57			sodium transporter
Morus005060	*NAT4*			-1.61	-1.84	Nucleobase-ascorbate transporter 4
Morus004830	*NAT1*		-1.00		-1.08	Nucleobase-ascorbate transporter 1
Morus005699	*LON1*		-1.22			Lon protease-like protein
Morus008688	*LIR1*		-1.14			hypothetical protein L484_008688
Morus013726	*MPT1*		-1.09			hypothetical protein L484_013726
Morus014474	*At2g33280*		-1.80			hypothetical protein L484_014474
Morus015285	*der*		-1.42			uncharacterized protein LOC21398388 isoform X1
Morus021239	*DTX33*		-1.48			MATE efflux family protein 9
Morus021318	*CHX20*		-1.23			Cation/H(+) antiporter 20
Morus021419	*PHT1-4*		-1.67			inorganic phosphate transporter 1–4 isoform X3
Morus023607	*ANTR3*		-1.81	-2.02	-1.98	probable anion transporter 3, chloroplastic
Morus023997	*At5g07050*		-1.88	-1.00		WAT1-related protein At5g07050 isoform X1
Morus004616	*At3g07010*			-1.12		probable pectate lyase 1
Morus004658	*PDR1*			-1.72		Pleiotropic drug resistance protein 1
Morus004807	*TTL4*			-1.05	-1.05	Inactive TPR repeat-containing thioredoxin
Morus014196	*Bp10*			-2.29	-2.53	L-ascorbate oxidase-like protein
Morus026138	*BOR2*			-1.11		putative boron transporter 2
Morus005142	*BOR4*				1.50	boron transporter 4
Morus007642	*POR1*				1.84	mitochondrial outer membrane protein porin of 36 kDa
Morus009648	*CCX1*				2.24	cation/calcium exchanger 1
Morus014617	*PLT6*				1.28	probable polyol transporter 6
Morus025438	*ABCG16*				2.61	ABC transporter G family member 16
Morus026124	*ABCB11*				1.50	ABC transporter B family member 11
Morus014211	*ABCG8*		1.04			ABC transporter G family member 8
Morus000158	*HEX6*				-1.55	Hexose carrier protein
Morus000166	*HEX6*				-1.04	Hexose carrier protein
Morus008545	*PUMP5*				-1.08	mitochondrial uncoupling protein 5
Morus009212	*STAR1*				-1.18	ABC transporter I family member 17
Morus012750	*ATHB-6*				-1.16	Homeobox-leucine zipper protein
Morus013409	*VATG*				-1.05	V-type proton ATPase subunit G
Morus019553	*NACK2*				-2.24	kinesin-like protein
Morus021142	*OCT4*				-1.14	organic cation/carnitine transporter 4
Morus021865	*RABC1*				-1.39	Ras-related protein

The DEGs analysis shows that several genes related to protein processing, mainly heat shock proteins (HSPs) were altered (Table 4). Surprisingly, the CK-vs-T0 group did not express any of these genes and the CK-vs-T2 group only expressed two of these genes. Meanwhile, moderate deficiency (CK-vs-T1) and high toxicity expressed many HSPs. For instance, in the CK-vs-T1, 22 DEGs of HSPs were expressed, whereas 21 DEGs were in CK-vs-T3. Curiously, all the HSPs genes were downregulated except Morus016474; *HSP22.0* and Morus025169; *HSP22.0*, encoding 16.9 kDa class I heat shock protein 1 (XP_010088689.1) and LOW-QUALITY PROTEIN: 22.0 kDa class IV heat shock protein (XP_024023540.1) were upregulated in CK-vs-T2 (Fig. 9B; Table 4).

**Table 4:** Differentially expressed genes related to protein processing

		CK-vs-T1	CK-vs-T2	CK-vs-T3	
ID	Symbol	log_2_(fc)	log_2_(fc)	log_2_(fc)	Description
Morus001270	*HSP23.6*	-1.24		-1.02	heat shock 22 kDa protein, mitochondrial
Morus005024	*HSP15.7*			-1.18	15.7 kDa heat shock protein, peroxisomal
Morus006034	*HSP18.5-C*	-1.66		-1.53	18.5 kDa class I heat shock protein
Morus013955	*HSP70*	-3.191		-1.97	heat shock 70 kDa protein
Morus012384	*HSP70-8*	-2.92		-2.03	heat shock 70 kDa protein 8
Morus018081	*HSP70*	-3.22		-2.05	heat shock 70 kDa protein
Morus016474	*HSP22.0*	-3.60	1.45	-2.31	16.9 kDa class I heat shock protein 1
Morus000338	*HSP17.4 A*	-3.43		-2.58	17.4 kDa class I heat shock protein
Morus006026	*HSP17.5-M*	-2.77		-2.58	17.5 kDa class I heat shock protein
Morus002575	*HSP21*	-3.74		-2.59	small heat shock protein, chloroplastic
Morus006031	*HSP17.5-M*	-3.35		-2.66	17.5 kDa class I heat shock protein
Morus006032	*HSP17.5-M*	-4.23		-2.76	kDa class I heat shock protein
Morus006023	*HSP17.3-B*	-3.37		-2.76	17.3 kDa class I heat shock protein
Morus025214	*HSP17.4 A*	-3.56		-2.87	17.4 kDa class I heat shock protein
Morus003779	*HSP17.9-D*	-2.64		-2.93	17.9 kDa class II heat shock protein
Morus025215	*HSP18.2*	-4.06		-3.05	17.5 kDa class I heat shock protein
Morus025169	*HSP22.0*	-4.32	1.45	-3.12	LOW QUALITY PROTEIN: 22.0 kDa class IV heat shock protein
Morus000756	*HSP17.5-M*	-4.48		-3.58	17.5 kDa class I heat shock protein
Morus018080	*HSP70*	-5.31		-4.10	heat shock 70 kDa protein 5
Morus003775	*HSP17.6*	-4.97		-5.77	17.3 kDa class II heat shock protein
Morus017688	*HSP18.1*	-15.10		-15.10	class I heat shock protein
Morus014800	*HSP1*	-1.04			Heat shock 70 kDa protein
Morus005860	*HSP18.2*	-1.76			17.4 kDa class I heat shock protein

**NOTE**: No heat shock genes were expressed in T0; hence, were not shown in the table

### Analysis of DEGs related to cell wall and transcription factor

Several DEGs related to plant cell wall were altered specifically in the Mn-treated leaves (Table 5). In the CK-vs-T1, four genes, including *PER19* (peroxidase 19), *XTH33* (putative xyloglucan endotransglucosylase/hydrolase protein 33), *HSP70* (heat shock 70 kDa protein) were downregulated, whereas *BXL1* (putative beta-D-xylosidase) was upregulated (Table 5). Meanwhile, 20 and 17 DEGs were observed in the CK-vs-T2 and CK-vs-T3, respectively, with most of the DEGs being downregulated. *At1g32860* (Glucan endo-1,3-beta-glucosidase 11), *XTH9* (endotransglucosylase/hydrolase protein 9), *XTH23* (endotransglucosylase/hydrolase protein 23), *XTHB* (probable xyloglucan endotransglucosylase/hydrolase protein B), etc., were altered and downregulated in CK-vs-T2 and CK-vs-T3. However, *XTH32* (putative xyloglucan endotransglucosylase/hydrolase protein 32), *RNS1*(extracellular ribonuclease LE), and *BXL1* were upregulated in CK-vs-T3 whereas *PECS-2.1* (putative pectinesterase/pectinesterase inhibitor 6), and *PME6* (pectinesterase) were upregulated in CK-vs-T2 (Table 5). Few transcription factors (TFs) were altered in response to the Mn treatments. In the CK-vs-T0, only one TF gene, *ERF6* (ethylene-responsive transcription factor 6) was upregulated (Table S3). In the CK-vs-T1, 8 TFs were observed. Among them, *BHLH87*, *NAC047*, *RAV1* (AP2/ERF and B3 domain-containing transcription factor) and *NAP2* (NAC transcription factor 29 isoform X1) were upregulated, whereas *WRKY72A*, *MYB111*, *NFYA1*, and *MYB28* were downregulated (Table S3). In the toxicity groups, 2 TFs (*MYB111* and *MYB28*) were altered in CK-vs-T2 and were downregulated whiles *NFYA1* (nuclear transcription factor Y subunit A-1 isoform X2) was downregulated and *MYB4*, upregulated in the CK-vs-T3 (Table S3).

**Table 5:** Differentially expressed genes related to plant cell wall

		CK-vs-T1	CK-vs-T2	CK-vs-T3	
ID	Symbol	log_2_(fc)	log_2_(fc)	log_2_(fc)	Description
Morus006981	*PEX1*		-1.35		Leucine-rich repeat extensin-like protein 2
Morus007711	*AED3*		-1.47		aspartyl protease
Morus008867	*PER19*	-1.22	-1.59	-2.56	peroxidase 19
Morus009426	*SBT1.7*		-1.20		subtilisin-like protease
Morus012273	*IMK3*		-1.17		Probably inactive leucine-rich repeat receptor-like protein kinase
Morus012731	*PHI-1*		-1.06	-2.55	protein EXORDIUM
Morus015371	*At1g32860*		-1.48	-1.05	Glucan endo-1,3-beta-glucosidase 11
Morus015671	*EXPA1*		-1.04		expansin-A15
Morus018309	*XTH9*		-1.80	-2.31	xyloglucan endotransglucosylase/hydrolase protein 9
Morus021491	*XTHB*		-1.57	-1.56	probable xyloglucan endotransglucosylase/hydrolase protein B
Morus022900	*XTH23*		-1.86	-1.88	probable xyloglucan endotransglucosylase/hydrolase protein 23
Morus022901	*XTH23*		-1.68	-2.38	probable xyloglucan endotransglucosylase/hydrolase protein 23
Morus025251	*EXPA6*		-1.44		expansin-A6
Morus027403	*SBT1.8*		-1.18	-1.21	subtilisin-like protease
Morus027795	*XTH33*	-1.30	-1.59	-1.98	putative xyloglucan endotransglucosylase/hydrolase protein 33
Morus007468	*CSLD5*		-1.12		cellulose synthase-like protein D5
Morus011995	*PME6*		1.05		pectinesterase
Morus011997	*PECS-2.1*		1.03		putative pectinesterase/pectinesterase inhibitor 6
Morus021541	*exgA*		-1.12		probable glucan 1,3-beta-glucosidase A
Morus001400	*TBL43*		-1.57		protein trichome birefringence-like 43
Morus020627	*XTH32*			1.52	putative xyloglucan endotransglucosylase/hydrolase protein 32
Morus015489	*LTPG1*			-1.33	non-specific lipid transfer protein GPI-anchored 1
Morus013955	*HSP70*	-3.19		-1.97	heat shock 70 kDa protein
Morus013081	*PER47*			-1.63	Peroxidase 47
Morus008516	*At5g34940*			-1.63	heparanase-like protein 3
Morus007739	*BXL1*	2.52		1.09	putative beta-D-xylosidase
Morus006991	*PME53*			-1.25	probable pectinesterase 53
Morus002150	*RNS1*			2.29	extracellular ribonuclease LE

**NOTE**: No cell wall genes were expressed in T0; hence, were not shown in the table

### Validation of the RNA-seq results by qRT-PCR

Validation of the RNA-Seq was performed on the 18 DEGs randomly selected for the qRT-PCR. From the results, 9 DEGs upregulated (*CAF1-11*, *AFS1*, *BHLH87*, *PME6*, *BGAL1*, *PECS-2.1*, etc.) in the RNA-Seq were confirmed to be upregulated by the qRT-PCR analysis (Fig. 9A). Similar results were observed for the downregulated genes (*SODCP*, *ANTR3*, *FSD2*, *HSP70*, etc.). A regression plot of the RNA-Seq and the qRT-PCR results showed a significant correlation coefficient (R^2^ = 0.8) exists between the RNA-Seq and the qRT-PCR results. The qRT-PCR results therefore confirm the reliability of the RNA-Seq result.

### Virus-induced gene silencing of the *Macax3* gene and the expression level in mulberry leaves after silencing

**Fig. 10:**
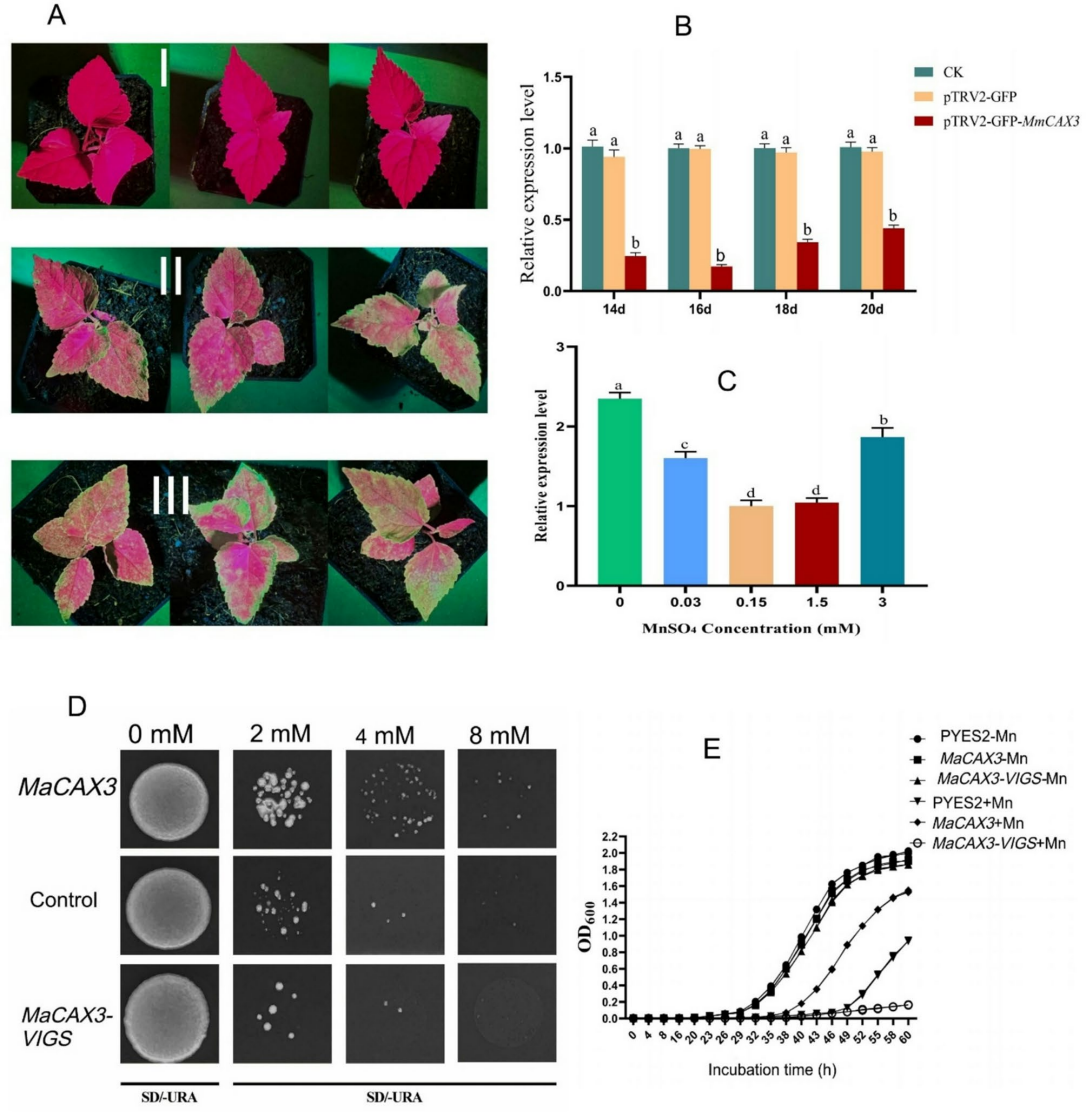
Functional validation of the mulberry *CAX3* gene. (**A**) Fluorescence detection results of mulberry leaves. (I) Blank control group; (II) pTRV2-GFP empty vector control group; (III) pTRV2-GFP-*MaCAX3* silencing vector infection group. (**B**) Relative expression level of *MaCAX3* gene after silencing. (**C**) Relative expression levels of *MaCAX3* gene under different concentrations of manganese treatment. (**D**) Heterologous expressions of *MaCAX3* and *Macax3*-VIGS in yeast treated with 0 mM Mn, 2 mM Mn, 4 mM Mn, and 8 mM Mn. Control indicates yeast cells transformed with the empty vector pYES2. *MaCAX3* indicates *MaCAX3*-overexpressing yeast. *Macax3*-VIGS indicates yeast that knocks down the *MaCAX3* gene expression. (**E**) Growth curve between the yeast expressing *MaCAX3* and *Macax3*-VIGS. -Mn indicates 0 mM Mn, +Mn indicates 4 mM Mn. Columns are the mean values of three replicates, and error bars represent the standard deviation of the three replicates. Different letters above the bars represent significant differences (Tukey’s HSD, *p* ≤ 0.05)

After the silencing of the mulberry *CAX3* gene (*MaCAX3*) by the VIGS technique, a hand-held fluorescence observation instrument was used to observe the fluorescence of mulberry leaves in the control group and the experimental group after infection. The results of the leaf fluorescence test showed that agrobacterium had successfully infected the mulberry seedlings (Fig. 10A). The relative expression level of *MaCAX3* gene in the blank control group and the empty carrier control group was basically the same (Fig. 10B), which excluded the influence of bacterial solution infection on the expression level of *MaCAX3*. In the silencing vector infection group, the relative expression level of *MaCAX3* decreased gradually from 14–16 d, and the silencing efficiency reached the highest on the 16th d. On the 18th to 20th d, the expression level of the *MaCAX3* gene increased, thus the silencing efficiency decreased. The results further showed that the silencing efficiency was best on the 16th d after infection. Again, the relative expression level of *MaCAX3* gene via qPCR in mulberry under 0, 0.03, 0.15, 1.5, and 3 mM MnSO_4_ treatments showed that the level of *MaCAX3* gene in mulberry was up-regulated under both Mn- deficiency (T0) and Mn-toxicity (T3) stress (Fig. 10C). Meanwhile the expression level of *MaCAX3* was only expressed and upregulated in the Mn-toxicity groups according to the transcriptome data.

### Heterologous expression of MaCAX3 in yeast and treated with different Mn concentrations

To understand the ability of MaCAX3 protein to transport Mn, the yeast expression vector of *MaCAX3*, *Macax3*-VIGS, and empty vector of *MaCAX3* and *Macax3*-VIGS were respectively transferred into BY4741 yeast. Positive strains were screened, and the bacilli were activated, diluted to the same volume, and cultured for 3 d on an SD-U solid medium containing 0 mM, 2 mM, 4 mM, and 8 mM MnSO_4_. The results reveal that in the medium without Mn, the size and number of yeast colonies containing the empty expression vector, *MaCAX3,* and *Macax3*-VIGS expression vector were basically the same, and the growth also remained the same (Fig. 10D). The yeast growth was inhibited in the 2 mM Mn treatment, but the growth of yeast expressing *MaCAX3* was least affected, and the growth of yeast expressing *Macax3*-VIGS was severely inhibited, which was more pronounced than that of yeast inverters containing the empty vectors. At 4 mM and 8 mM Mn treatment, the yeast growth was basically the same as in the 2 mM Mn treatment and the trend was more obvious. The results showed that under the condition of Mn treatment, the heterologous expression of *MaCAX3* increased the transport of Mn in yeast, thus inhibiting the toxic effect of Mn, and the yeast *Macax3*-VIGS that knocked down *MaCAX3* gene was more seriously poisoned by Mn than the yeast that expressed the *MaCAX3* gene, indicating that *MaCAX3* has a strong ability to transport Mn.

### Growth curve under yeast-converted seeds with different Mn treatments

To further understand the effect of *MaCAX3* and *Macax3*-VIGS expressions on the Mn capacity of the yeast, yeast translators containing the empty vector, *MaCAX3,* and *Macax3*-VIGS gene expression vector were cultured overnight. The same bacterial solution was inoculated into an SD-U liquid medium containing 4 mM Mn and those without Mn and cultured for 60 h, and the growth curve was measured. From the results (Fig. 10E), in the absence of Mn, the growth of the empty carrier yeast and the yeast expressing *MaCAX3* and *MaCAX3*-VIGS genes was consistent within 0 h to 20 h, and after 20 h, the OD_600_ values of the three treatments showed exponential growth with an increase in time. There was no difference in the growth rate among the three yeast treatments. When 4 mM Mn was added, the growth of empty carrier yeast was consistent with that of yeast expressing *MaCAX3* and *Macax3*-VIGS genes within 0 h to 35 h, and after 35 h, the OD_600_ value of the yeast expressing *MaCAX3* gene began to show an exponential increase. The OD_600_ value of the yeast containing the empty vector began at 43 h. The yeast expressing the *Macax3*-VIGS gene grew slowly after 49 h of culture. The OD_600_ value reached about 0.16 after 60 h of culture. The above results showed that the growth of yeast expressing *Macax3*-VIGS gene was poor compared to that of yeast inverters expressing *MaCAX3* gene under Mn treatment. The experimental results of the growth curve of yeast inverters treated with Mn were consistent with the results of the point plate experiment, and both showed that the yeast expressing *Macax3*-VIGS gene was more severely inhibited than the yeast expressing *MaCAX3* gene under Mn stress (Fig. 10D, E). These results suggest that *MaCAX3* gene plays an important role in mulberry Mn tolerance.

## Discussion

### Mn toxicity induces higher DEGs implicated in Mn transport and detoxification in mulberry plants relative to deficiency

The plant systems demand an optimal level of metal nutrients, including Mn for normal growth and development, and to maintain their homeostasis. To counteract the effects of Mn stresses, the acquisition from the rhizosphere and distribution of Mn within the plant cells must be regulated to ensure fine-tuned uptake and translocation in the plant cells. The mechanistic regulation of Mn acquisition, distribution, and translocation from the soil, which is coordinated by plant-specific transporters, is one of the evolved mechanisms employed by plants to ensure Mn distribution to other parts of the plant and to avoid hyperaccumulation via detoxification. This current study identified several DEGs associated with transporters and detoxification, which are possibly involved in Mn acquisition, translocation, distribution, remobilization, and sequestration in mulberry plants exposed to different levels of Mn. These genes were not only identified to be implicated in Mn transport and detoxification, but the functional validation and characterization of *CAX3* gene (vacuolar cation/proton exchanger 3) support the hypothesis that the genes identified in our study are crucially involved in Mn transport and detoxification (Fig. 10; Table 3). Intriguingly, *CAX3* was identified as both mulberry transporter and detoxification protein (Tables 2 and 3**)**. The cation/H^+^ exchanger has been identified and functionally characterized to play important roles in the vacuolar accumulation of heavy metals, including Mn [15]. A recent study reported that the overexpression of *CAX3* improves cadmium (Cd) tolerance by minimizing ROS production through the activation of Ca levels in Arabidopsis. *CAX* genes are reported to be implicated in the sequestration of metal ions such as Mn, lithium (Li), Cd, and calcium (Ca) from the cytosol into the vacuole for possible recycling, efflux, and detoxification [15]. Additionally, the Arabidopsis *CAX2* gene (*AtCAX2*) was directly involved in conferring tolerance to Mn toxicity when expressed in a Mn-sensitive yeast mutant pmc1vcx1cnb or in tobacco via sequestration of Mn into vacuoles [29]. In this study, the upregulation of *CAX3* gene expressing exclusively in Mn toxicity treatments (CK-vs-T2 and CK-vs-T3) but not in CK-vs-T0 and CK-vs-T1, highlights its crucial role in the vacuolar accumulation of Mn for final detoxification. To affirm the above development via functional validation and characterization of *CAX3*, a heterologous expression of *MaCAX3* in yeast treated with different levels of Mn was performed to elucidate the mechanism that, MaCAX3 protein is indeed involved in the transport of Mn. The validation study revealed that under higher Mn treatments (4 mM and 8 mM), the heterologous expression of *MaCAX3* gene significantly elevated the yeast growth in the Mn, signaling the inhibition of Mn (Fig. 10D). Intriguingly, the yeast growth of the *macax3-VIGS* that silenced *MaCAX3* gene was found to be more disrupted and damaged by Mn relative to the yeast expressing the *MaCAX3* gene (Fig. 10D), highlighting the involvement of *MaCAX3* in the transport of Mn. A knockout study revealed that the *Arabidopsis thaliana* cation transporter (*AtCAX2*) not only plays a major physiological role in Ca homeostasis but is also involved in vacuolar Mn accumulation [30]. Meanwhile, their study further reported that vacuolar Mn^2+^ transport was not completely inhibited in *cax2*. Our current study observed similar patterns of activity in the silenced gene, where some activities of Mn in yeast were observed, with transient expression of *MaCAX3* in different Mn levels being recorded (Fig. 10E).

What is fascinating about Mn transport and distribution across membranes is that most of these proteins involved in Mn transport are unspecific for the metal. The plant Mn transporters have been found to transport not only Mn but also other divalent cations, including Fe, Zn, Cu, Cd, Ca, Co, and Ni [31]. Our findings revealed other transporter genes putatively involved in the transport of Mn and other elements. The significant upregulation of transporter genes, including boron transporter 4 (*BOR4*), mitochondrial outer membrane protein porin of 36 kDa (*POR1*), cation/calcium exchanger 1 (*CCX1*), probable polyol transporter 6 (*PLT6*), ABC transporter G family member 16 (*ABCG16*), ABC transporter B family member 11 (*ABCB11*), sulfate transporter 2.1 (*SULTR2;1*), protein NRT1/ PTR FAMILY 4.3 (*NPF4.3*) and downregulation of putative sulfate transporter 3.4 (*SULTR3;4*), protein NRT1/ PTR FAMILY 5.1 (*NPF5.1*), putative boron transporter 2 (*BOR2*) etc., under excess Mn treatments (Table 3), may highlight their possible roles in Mn transport, acquisition, translocation, distribution, and possible influx to the vacuole for sequestration during Mn toxicity. To buttress our findings, homologs of ATP-binding cassette (*ABC*) transporters were reported to be regulated in stylo plants subjected to Mn toxicity and implicated in the transport of hormones, secondary metabolites, and heavy metal ions [2]. Interestingly, the *CCX1* gene, which was found to be upregulated in this study, was reported to be downregulated under both Al and Mn toxicity in wheat plants [32], suggesting the differential genetic variations in plant’s response to metal toxicity. On the contrary, Mn-deficient-induced mulberry leaves altered fewer DEGs involved in Mn transport and detoxification. Among these transporter genes include serine/threonine exchanger SteT (*At3g13620*), peroxisomal membrane protein (*PMP22*), and sodC protein (*SODCP*) and were downregulated (Fig. 9D; Table 3), indicating lower Mn transporter activities under lack of Mn treatment. Under Mn deficiency, only the U-box domain-containing protein 18 (*PUB18*) was upregulated. *PUB18* is crucial in the regulation of abscisic acid (ABA) and mediates the movements of stomatal [33].

Plants have evolved versatile detoxification mechanisms to counteract the phytotoxicity of heavy metals. These have been achieved via coordinated regulation and activation of enzymes, genes, and proteins involved in the chemical modification in plants. For example, the protein detoxification gene functions as xenobiotic transmembrane transporter, antiporter activity, and xenobiotic detoxification by transmembrane export across the plasma membrane. In our study, two detoxification genes, *DTX49* and *DTX14* were up- and downregulated, respectively, in CK-vs-T1 and CK-vs-T3 (Table 3). The regulation of these genes under Mn treatments indicates their role in Mn detoxification and sequestration in mulberry. The detoxification protein (*DTX*), a.k.a Multidrug and toxic compound extrusion transporter (MATE), has recently been identified and characterized in *Arabidopsis* and found to be implicated in detoxification of toxic compounds and heavy metals, tolerance to aluminum toxicity, iron homeostasis, and disease resistance [34]. Additionally, other genes involved in detoxification of metals, including copper chaperone for superoxide dismutase (*CCS*), *SODCP* (in T0) and peroxidase 19 (*PER19*), peroxidase 47 (*PER47*) and protein Fe superoxide dismutase 1 (*FSD3*) (in T3) were all exclusively downregulated (Fig. 9D; Tables 2 and 3). This means that mulberry plants exposed to Mn stress adopt downregulation mechanisms to ensure Mn tolerance. It could be concluded that mulberry plants exposed to toxic Mn stress significantly induces higher DEGs involved in transport and detoxification relative to deficiency, suggesting the enormity of cell disruption and burst caused by Mn toxicity.

### The exposure of mulberry to Mn triggers the regulation of DEGs involved in antioxidant, cell wall, and defense

Plants under Mn stress produce a higher amount of ROS that causes oxidative burst, damaging and disrupting the cell structure [1]. Plants can curb the ripple effects of ROS via the activation and synthesis of antioxidants and ROS-scavenging enzymes. Glutathione S-transferase (GST) is a widely known plant metabolite that acts as an antioxidant and detoxifier, a known precursor to phytochelatin, and chelation of heavy metals, including lead (Pb), arsenic (As), mercury (Hg), Cd, Ca, Mn, and Al. The GST performs the chelation mechanism by binding the heavy metal ions to the sulfhydryl group of enzyme proteins, thereby reducing thier bioavailability capabilities for plant acquisition [35]. Furthermore, GST acts as a direct precursor to phytochelatin peptides using a synthase enzyme to form complex-free ions that are transported to the plant vacuole for final detoxification [36]. A recent report [37] on mulberry plants exposed to Cd stress markedly increased the expression of genes (10) implicated in the GST metabolic pathway in roots, stems, and leaves. In this study, two GST genes, including glutathione S-transferase (*GSTF8*) and probable phospholipid hydroperoxide glutathione peroxidase (*CSA*) were exclusively downregulated in Mn toxicity treatment but failed to express in the deficiency (Fig. 9C; Table 2). The reduced expression and lower accumulation of GSH or GSSG (reduced and oxidized) could be a result of disrupted sulphur absorption in plants under Mn stress. The mere expression of these genes under Mn toxicity could suggest a crucial role played by GST, which acts as a metal detoxification enzyme to protect plants from oxidative damage and confers Mn tolerance in plants. Our finding is in contradiction with those reports [2], where homologs to glutathione S-transferase (GST) were significantly upregulated by Mn toxicity in stylo plants. To further affirm our results, ROS and total antioxidant capacity (TAC) under Mn toxicity were markedly elevated (Fig. 1D, E) relative to other treatments, indicating the presence of ROS production, and the activation of antioxidant systems to counteract oxidative burst and membrane damage during Mn toxicity in mulberry leaves. Intriguingly, Mn deficiency triggered two interesting antioxidant genes *PPO* encoding polyphenol oxidase (Morus002312 and Morus012124), which were up- and downregulated, respectively (Table 2). The significant upregulation of *PPO* under Mn deficiency was further exemplified and supported by the higher PPO activity observed under extreme Mn deficiency in mulberry plants (Fig. 1C). The expression of these genes exclusively under Mn deficiency (and not in Mn treatments) underscores the fact that PPO not only counteracts oxidative damage caused by ROS but also plays a crucial role and acts as a defense barrier in plants. Notably, we found that all the antioxidant genes induced under Mn toxicity were mostly downregulated, highlighting that mulberry plants exposed to Mn toxicity utilize downregulation mechanisms to demystify the effect of excess ROS production. Furthermore, antioxidant genes encoding two L-ascorbate peroxidase 2 *APX2* (Morus001808 *and* Morus001810), and isogenes of peroxidases (peroxidase 19, peroxidase 47, cationic peroxidase 2), protein Fe superoxide dismutase 1, superoxide dismutase [Fe], and probable phospholipid hydroperoxide glutathione peroxidase) were downregulated under excessive Mn supply (Table 2). The excess Mn regulation of APX-related transcripts resulted in increasing the lipid peroxidase, hydrogen peroxide, and hydroxyl radical scavenging activities in mulberry leaves subjected to excess Mn toxicity (Fig. 1A, B & F), suggesting that these antioxidant enzymes and genes play a leading role in ROS scavenging in mulberry. This is consistent with our previous study that reported increased activity of peroxidase (POD) and superoxide dismutase (SOD) in mulberry under Mn deficiency and toxicity in comparison to the control [1]. However, our finding is at odd with a previous study reporting that homologous genes encoding antioxidant enzymes were upregulated in stylo and citrus (*Citrus grandis*) in response to Mn toxicity [6, 38]. This contradictory finding is underscored by the fact that different plant species may employ varied mechanisms to curtail excess ROS production, increasing ROS scavenging activity and conferring Mn tolerance. The peroxidase 21 (*PER21*) gene was identified to be upregulated under moderate deficiency (Table 2), suggesting a crucial role played in ROS scavenging under Mn supply.

Emerging research findings have identified heat shock proteins (HSPs) to be implicated in both oxidation, defense and protein processing [39]. Our findings identified a series of HSPs categorized as oxidation, and defense-response genes encoding diverse antioxidant enzymes (Tables 2 and 4). Most of these genes were expressed under the Mn toxicity and were downregulated, except 16.9 kDa class I heat shock protein 1; *HSP22.0* (Morus016474) and 22.0 kDa class IV heat shock protein; *HSP22.0* (Morus025169) (Table 4), which were upregulated under toxicity (T2). Heat shock proteins (HSPs) confer biotic and abiotic tolerance to plants under stress. HSPs improve membrane stability and detoxify ROS by modulating and regulating the antioxidant systems in plants under stress. Notable among these HSPs identified in this study include *HSP23.6*,* HSP15.7*,* HSP18.5-C*,* HSP70*,* HSP70-8*, etc., and were all downregulated (Fig. 9B; Table 4). The very reason for the induction of these higher numbers of HSP genes under Mn treatments in mulberry is quite novel and the rationale behind the downregulation of these HSP genes remains unanswered and requires further confirmation. The regulation and expression of these HSPs could suggest improvement in membrane stability and detoxification of ROS by inducing higher antioxidant-related genes and enzyme activities under Mn toxicity in mulberry. However, the lack of expression of these HSPs under deficiency treatment highlights the fact that membrane stability was not severely compromised, and no detoxification activity of excess Mn was carried out in that regard. Similarly, these heat shock proteins (HSPs) were identified together with other defense proteins in stylo plants exposed to Mn toxicity [2].

A recent study conducted in wheat found that genes involved in cell wall regulation and modification were specifically triggered exclusively in Al treatments but not under Mn supply [32]. In our study, 28 genes were altered in response to Mn treatments (Table 5) and were identified to be involved in cell wall regulation and biosynthesis contrary to what was reported in wheat plants [32]. These contradictions may not only result from variations in species genetic make-up but also due to differential mechanisms employed by diverse species to cope with heavy metal stresses, including Mn. Interestingly, all the cell wall biosynthesis genes were only expressed in Mn-treated leaves compared to the lack of Mn-treated leaves. Xyloglucan endotransglucosylases (XTHs) are crucial enzymes that govern the strength and ductility of the cell wall and are found to be extremely sensitive to metals, especially Al [40]. Whilst Luo et al. identified varied differential expression patterns of XTHs in wheat, with genes such as *XTH22*, *XTH23*, *XTH25*, *XTH27*, and *XTH28* observing upregulation patterns and *XTH8*, *XTH16*, *XTH24*, *XTH26*, *XTH30*, and *XTH31* also observing downregulation under Al stress [32]. However, we identified *XTH*s genes including *XTH9*,* XTHB*,* XTH23*,* XTH33*, and *XTH32* to be exclusively downregulated in Mn-treated leaves (Table 5). Several *XTH* genes were reported in the mulberry genome when the plant was subjected to magnesium stress [41, 42]. Plants under stress confer tolerance by decreasing the accumulation of xyloglucans through the downregulation of the *XTH* genes [40]. This phenomenon suggests that mulberry plants tolerate Mn-toxicity stress by reducing the biosynthesis of xyloglucans through the downregulation of *XTH* genes. This finding was further confirmed by the reduction in cellulose, hemicellulose, and lignin contents observed under Mn toxicity in mulberry in this study (Fig. 2B-D). The reduction in cellulose, hemicellulose, and lignin contents concomitantly reduced cellulase activity under Mn toxicity in mulberry (Table 1). Similarly, the activity of XTH in response to Al treatments directly repressed the activity of XTH enzyme within 30 min of supply and was reported to have triggered the concomitant deposition of callose in roots [40]. Additionally, *CSLD5* (cellulose synthase-like protein) was not only downregulated and exclusively expressed in Mn toxicity treatments (Table 5), but the activity of cellulase (Table 1) and cellulose content (Fig. 2B) were markedly reduced, respectively, in response to Mn toxicity. This hypothesis suggests that cell wall polysaccharides of mulberry are reduced in response to excess Mn and consequently affect gene expressions, enzyme activities, and metabolite content to tolerate Mn stress. Another cell wall polysaccharides gene encoding pectinesterase was expressed in this study. We identified three pectinesterase genes, including *PME6* (pectinesterase), and *PECS-2.1* (putative pectinesterase/pectinesterase inhibitor 6) which were significantly upregulated and *PME53* (probable pectinesterase 53) gene observing downregulated in Mn toxicity (Table 5). The upregulation of *PME6* and *PECS-2.1* correlates with the high pectin content recorded under Mn toxicity (Fig. 2E), highlighting the crucial role of pectin biosynthesis during Mn stress. Meanwhile, the activity of pectinase was lower and corroborates with the downregulation of the *PME53* gene in Mn toxicity (Tables 1 and 5). The level of consistency observed in the results of this study confirm the accuracy and reliability of our results. Mn stress not only significantly increases the contents of cell wall polysaccharides such as pectin, and the activities of pectinase, but also elevates the cell wall-related genes in leaves of mulberry. The presence of pectin, cellulose, and hemicellulose in plant cell walls plays a key role in chelating and binding to heavy metals and it has been reported that about 85% of Cd are sequestered in the pectins, cellulose, and hemicellulose fractions of the cell wall to lessen metal poisoning [43]. We can speculate that the upregulation of genes involved in cell walls and the higher contents of pectin observed in our study suggest that the presence and accumulation of pectin and other polysaccharides may sequester and detoxify the higher amount of Mn in mulberry. Interestingly, none of the plant cell wall-related genes and transcripts were identified under Mn deficiency but the contents and activities of cell wall metabolites and enzymes increased, highlighting less disruption to membranes.

## Conclusion

Putting together, we conclude that treatment of mulberry with Mn not only triggered the downregulation of genes involved in transport and detoxification but also altered the downregulation of genes implicated in antioxidants, oxidation, defense and cell wall, highlighting that mulberry generally utilizes downregulation mechanisms to retrench Mn stress. Mn toxicity significantly induced a higher number of DEGs relative to deficiency, suggesting the complex mechanisms employed by mulberry plants to cope with and tolerate Mn stresses. Mn supply, in parts, observed differential and divergent regulation and activity patterns in the genes, metabolites, and enzymes related to oxidation, antioxidants, and cell walls. The accumulation of metabolites, including pectins, cellulose, hemicellulose, and lignin, and the activation of enzymes and genes, indicate that mulberry plants sequestered excess Mn via complex formation and chelation.

## Electronic supplementary material

Below is the link to the electronic supplementary material.

Supplementary Material 1

Supplementary Material 2

Supplementary Material 3

Supplementary Material 4

## Data Availability

Data used in this work are described in the article, and others are attached as supplementary materials. Transcriptome raw data with the accession number PRJNA1217365 was deposited in the NCBI Sequence Read Archive (SRA) and is publicly available via the link provided below: (https://www.ncbi.nlm.nih.gov/search/all/?term=PRJNA1217365).
