# Supplementary material for: Transcriptome-based analysis reveals key molecular mechanisms and functional characterization of MaCAX3 gene involved in manganese stress responses in mulberry plants
Source: BMC Plant Biol. 2025 Jul 29;25:971. doi: 10.1186/s12870-025-06767-5 (PMC12305966; doi:10.1186/s12870-025-06767-5)
Supplement: Supplementary file 1 — Supplementary Material 1 [file 12870_2025_6767_MOESM1_ESM.docx]

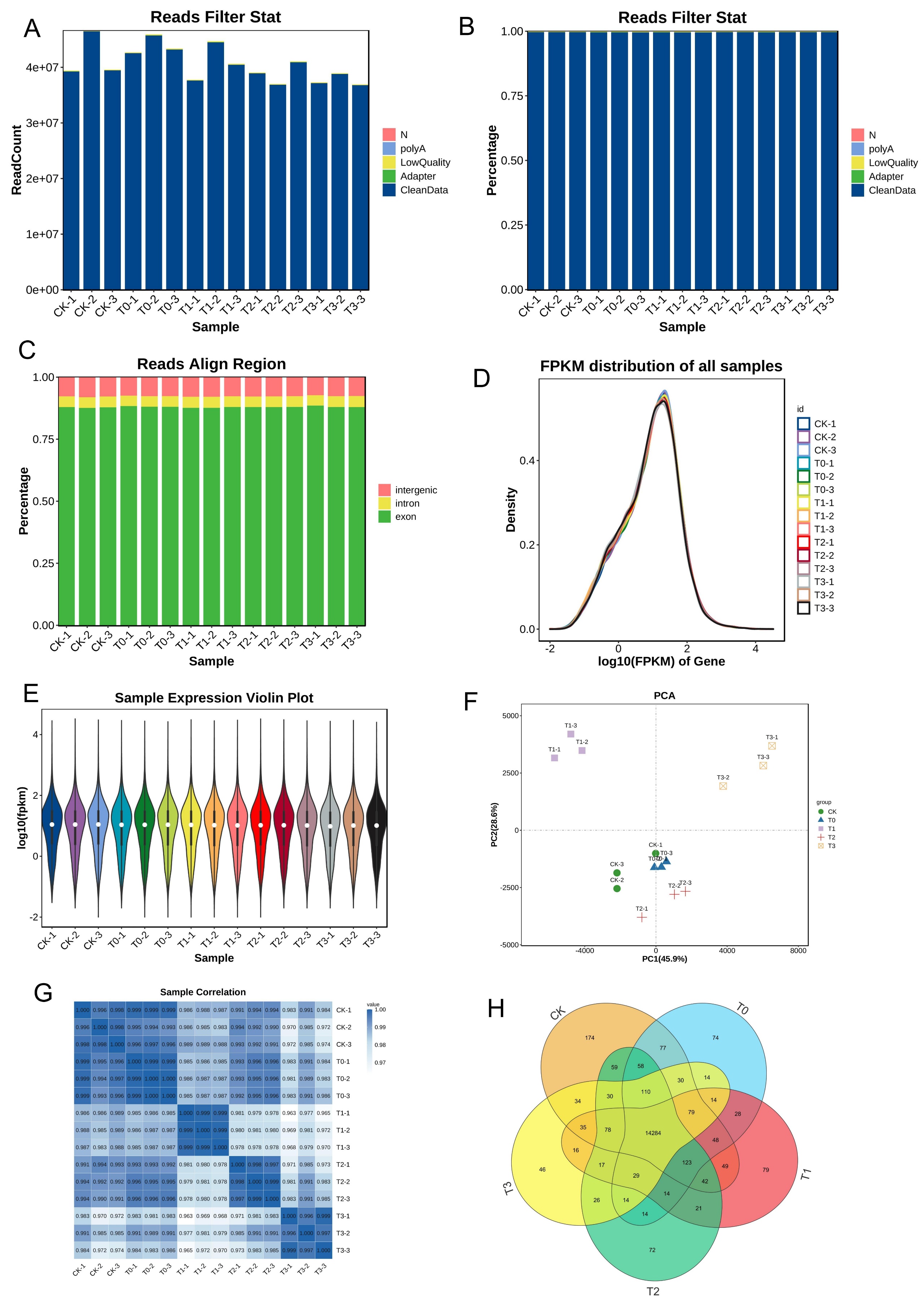


**Fig. S1** Mulberry transcriptome profile statistics . (A) Reads count filtering. (B) Read filtering (%). (C) Reads regional alignment (%). (D) Density plot of FPKM. (E) Sample violin expression plot (F) Principal component (PC) analysis. (G) Sample correlation heatmap analysis. (H) Venn diagram of treatment gene expression in mulberry plants exposed to Mn deficiency (T0), moderate deficiency (T1), sufficiency (CK), moderate toxicity (T2) and toxicity (T3).


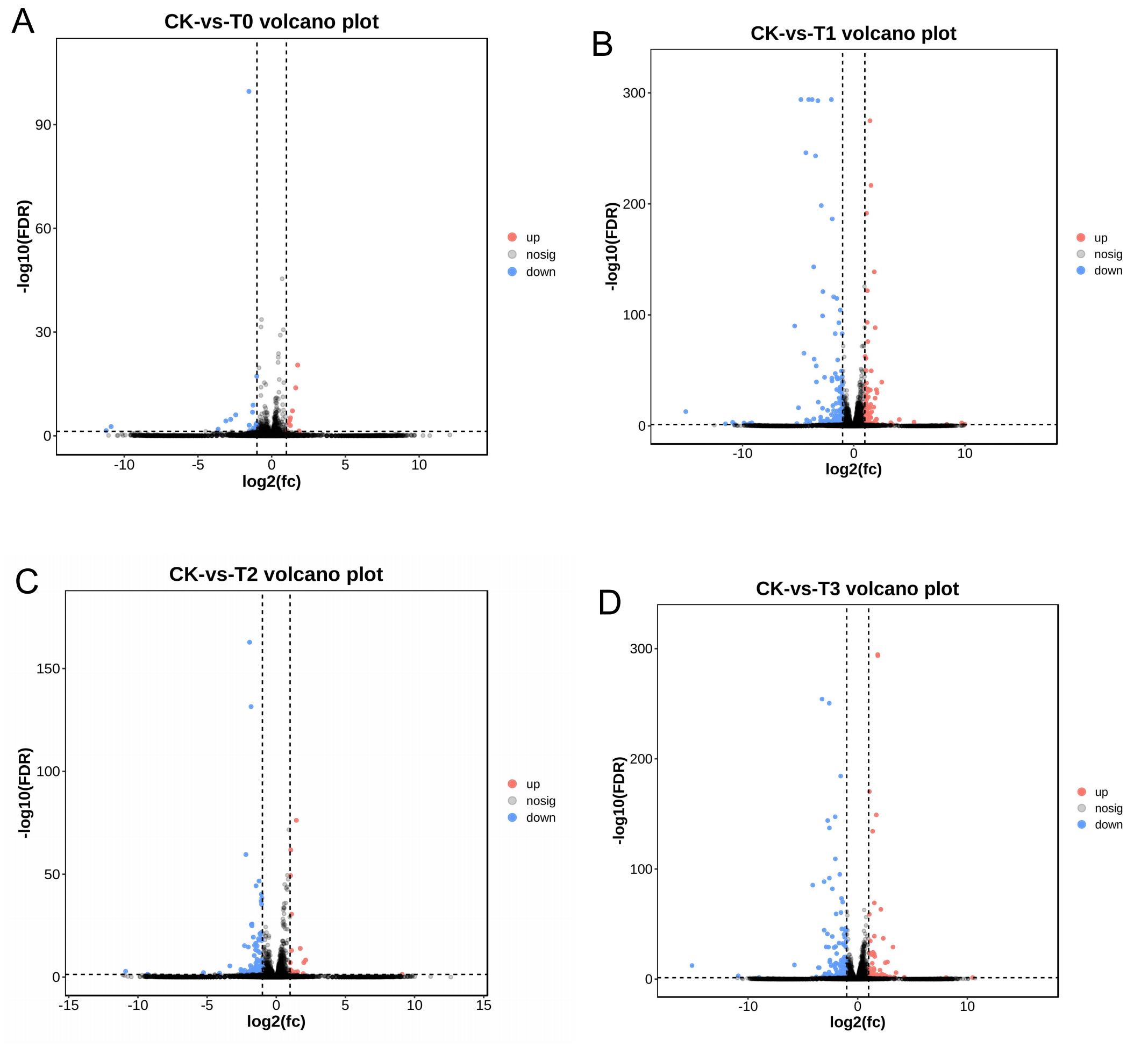


Fig. S2. Volcano plot of the DEGs in Mn deficiency (A), moderate deficiency (B), moderate toxicity (C) and toxicity (D). Red dot represents upregulated genes and blue dot represents downregulated genes.


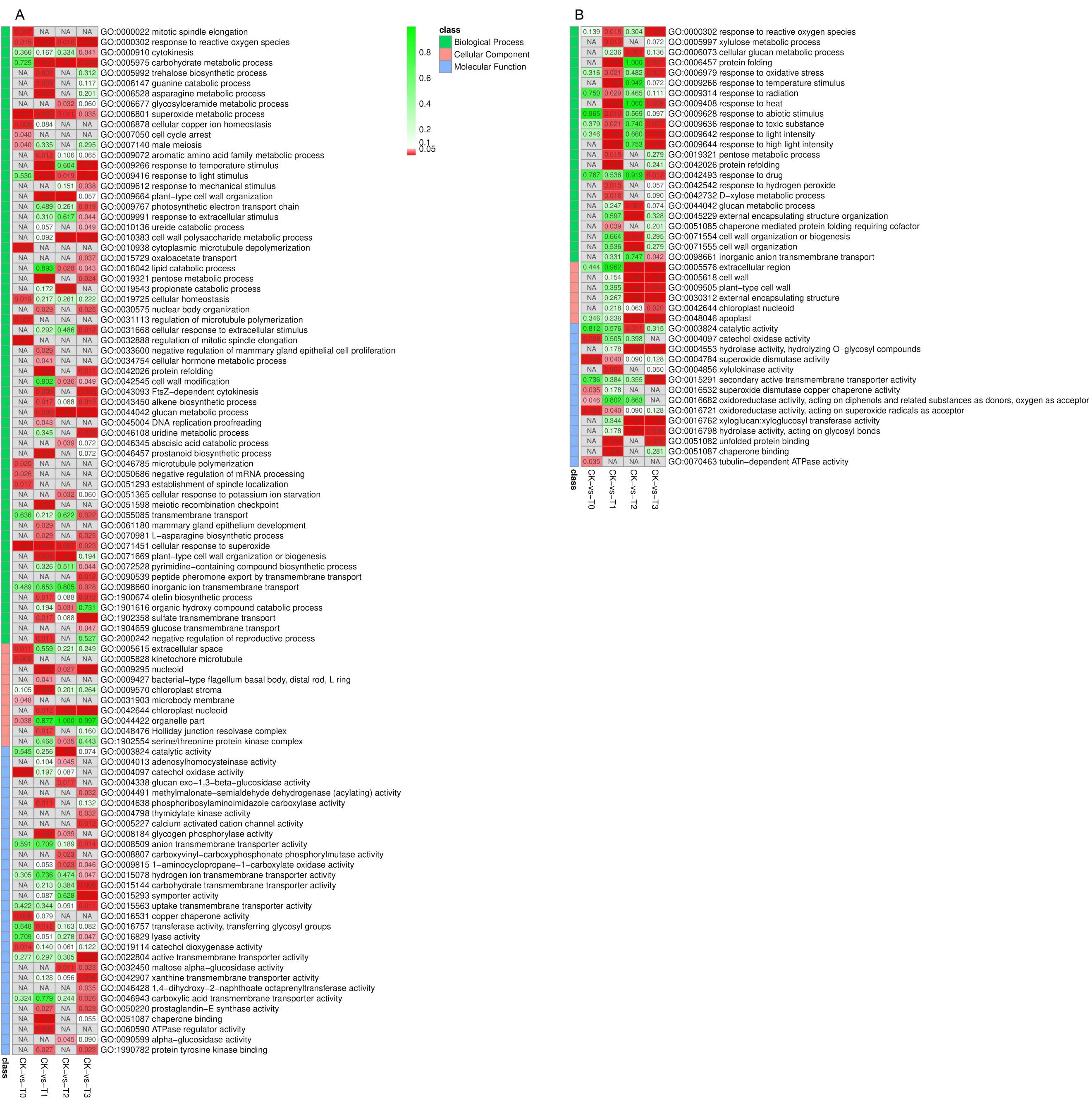


**Fig. S3** Heatmap of Gene ontology (GO) functional annotation. (A) GO term classification analysis based on p-value. (B) GO term classification analysis based on corrected p-value (FDR≤0.05). The colors represent the concentration of DEGs. T0, T1, are Mn deficiency group. T2 and T3 are Mn toxicity treatments. CK is the sufficient group.
